# Investigating the influence of maternal prenatal BMI and perinatal depressive symptoms on neonatal brain network dynamics

**DOI:** 10.1038/s41390-025-04726-2

**Published:** 2026-01-15

**Authors:** Isabella L. C. Mariani Wigley, Alexandra Lautarescu, Elena Vartiainen, Elmo P. Pulli, Niloofar Hashempour, Harri Merisaari, Wajiha Bano, Silja Luotonen, Ashmeet Jolly, Ilkka Suuronen, Linnea Karlsson, Hasse Karlsson, Joana Cabral, Morten L. Kringelbach, Dafnis Batalle, A. David Edwards, Jetro J. Tuulari

**Affiliations:** 1 FinnBrain Birth Cohort Study, Turku Brain and Mind Center, Department of Clinical Medicine, University of Turku and Turku University Hospital, Turku, Finland; 2 Centre for Population Health Research, Turku University Hospital and University of Turku, Turku, Finland; 3 Department of Forensic and Neurodevelopmental Sciences, Institute of Psychiatry, Psychology & Neuroscience, King’s College London, London, UK; 4 Early Life Imaging Research Department, School of Biomedical Engineering and Imaging Sciences, King’s College London, London, UK; 5 Department of Pediatric Neurology, Turku University Hospital, Turku, Finland; 6 Department of Psychology and Speech-Language Pathology, University of Turku, Turku, Finland; 7 Department of Teacher Education, University of Turku, Turku, Finland; 8 Department of Child Psychiatry, University of Turku and Turku University Hospital, Turku, Finland; 9 Department of Clinical Medicine, Unit of Public Health, University of Turku, Turku, Finland; 10 Department of Psychiatry, University of Turku and Turku University Hospital, Turku, Finland; 11 Centre for Eudaimonia and Human Flourishing, Linacre College, University of Oxford, Oxford, UK; 12 Department of Bioengineering and Institute of Systems and Robotics, LARSyS - Laboratory for Robotics and Engineering Systems, Instituto Superior Tecnico, University of Lisbon, Lisbon, Portugal; 13 Centre for Music in the Brain, Department of Clinical Medicine, Aarhus University, Aarhus, Denmark; 14 Department of Psychiatry, University of Oxford, Oxford, UK; 15 MRC Centre for Neurodevelopmental Disorders, King’s College London, London, UK; 16 Clinical Neurosciences, University of Turku and Turku University Hospital, Turku, Finland; 17 Neurocenter, Turku University Hospital, Turku, Finland

## Abstract

**Background:**

Elevated pre-pregnancy body mass index (BMI) and perinatal depressive symptoms have been linked to neonatal alterations in brain structure and function. This study examined associations between neonatal functional brain dynamics, maternal BMI, and perinatal depressive symptoms measured by the Edinburgh Postnatal Depression Scale (EPDS) in a community-based, largely low-risk cohort.

**Methods:**

Funcitonal MRI and Leading Eigenvector Analysis (LEiDA) were applied in a neonatal cohort (*N* = 437; 236 males; mean gestational age 39.6 weeks) from the developing Human Connectome Project. We assessed whether neonatal brain-state probabilities related to maternal BMI and EPDS scores (*M* = 5.6, SD = 4.3), testing main effects and, separately, their interaction. The sample included 291 healthy-weight (BMI < 25), 98 overweight (25 BMI < 30), and 48 obese (BMI 30) mothers.

**Results:**

EPDS scores were low in this cohort and did not demonstrate associations with brain states or a significant BMI × EPDS interaction. Higher maternal pre-pregnancy BMI was negatively associated with the stability of a functional network encompassing superior frontal, superior parietal, and temporal regions (*ß* = −0.129, *p* = 0.006).

**Conclusion:**

As this network is normally recruited more with age, reduced stability suggests slowed maturation of fronto-parieto-temporal systems and may signal early risk for later behavioral challenges.

**Impact:**

- Higher maternal pre-pregnancy BMI is associated with reduced stability in a neonatal frontoparietal brain state, characterized by coordinated activity in frontal, parietal, and temporal regions.
- This state is one of six distinct dynamic connectivity patterns identified, reflecting core neonatal resting-state networks.
- The association was robust across multiple analytic models and clustering solutions.
- No significant effects were found for maternal depressive symptoms.
- These findings underscore the selective impact of maternal metabolic health on early brain organization, suggesting prenatal influences on the functional architecture of the newborn brain that may shape long-term neurodevelopmental trajectories.

## Introduction

Researchers have long been intrigued by the complex relationship between maternal well-being during pregnancy and its potentially profound implications for fetal health and long-term outcomes.^1^ Guided by the principles of the Developmental Origins of Health and Disease and Fetal Programming, our understanding of how prenatal environments shape future health trajectories has advanced considerably.^2,3^ These theories suggest that environmental exposure during critical periods of fetal development can have lasting effects on various physiological systems, including brain development, influencing disease risk later in life.

In this context, maternal physical well-being during pregnancy, and in particular overweight and obesity, have been associated with long-term neurodevelopmental outcomes in children with research suggesting that they may impact offspring brain development in utero. Previous studies have highlighted both structural and functional alterations in infants born to overweight mothers, as well as BMI-related neural phenotypes.^4–6^ For instance, one study found that higher maternal prepregnancy BMI relates to lower cortical thickness in key regions of the frontal lobe (i.e., left pars opercularis gyrus, left pars triangularis gyrus, and left rostral middle frontal gyrus).^7^ Moreover, changes in functional connectivity associated with reward processing and cognitive control, like those observed in obese adults, were also found in 2-week-old infants born to mothers with high BMI.^8,9^ Infants born to healthy-weight mothers showed a greater recruitment of the dorsal anterior cingulate cortex regions into the prefrontal network compared to infants born to overweight mothers; functional connectivity strength in the dorsal anterior cingulate cortex was negatively correlated with maternal fat mass percentage measured in early pregnancy.^8^ This evidence suggests that exposure to maternal obesity in utero may be linked to changes in resting-state functional connectivity in newborns.

Beyond weight, other aspects of maternal well-being during pregnancy may influence offspring brain development. For instance, depressive symptoms during pregnancy have been linked to poorer neurobehavioral outcomes in children as well as alterations in brain structure and function.^10–12^ Those include, in neonates born to mothers with higher levels of prenatal depressive symptoms, altered diffusion properties in the amygdala,^13^ increased diffusivity in right frontal regions,^14^ decreased structural connectivity between the right amygdala and the right ventral prefrontal cortex,^15^ and increased functional connectivity of the amygdala with the left temporal cortex and insula, the bilateral anterior cingulate, medial orbitofrontal, and ventromedial prefrontal cortices.^16^ As evident from the above, numerous studies have investigated the link between exposure to maternal perinatal depressive symptoms and brain development in children. However, the specific brain regions involved and the direction of these associations remain inconsistent. Models of dynamic functional connectivity, which characterize functional networks over time, could help elucidate these effects by providing new insights into how maternal depressive symptoms alter neural development and connectivity patterns in the fetal brain under suboptimal conditions. Indeed, a recent study using resting-state functional magnetic resonance imaging (rs-fMRI) and a small sample of 20 neonates reported an association between maternal prenatal distress and the stability of a fronto-parietal brain network.^17^

Rs-fMRI is a widely used technique for investigating the interactions between brain regions and mapping comprehensive whole-brain functional connectivity networks. Brain connectivity is inherently dynamic and characterized by non-stationarity, with several models of dynamic functional connectivity (dFC) proposed to capture this variability.^18^ These methods track the real-time neural adjustments needed to regulate various brain states, adapt to transient conditions, and integrate information in order to study the formation, interaction, and dissolution of these network configurations over time.^19^ While the sliding window approach has been the most common method for evaluating dFC,^20^ other methods with higher temporal resolution are gaining recognition for their ability to produce relevant and meaningful results. These methods include co-activation pattern analysis^21^ and phase-coherence pattern analysis.^22^ The former examines frames where regional activity exceeds a certain threshold, while the latter focuses on phase-locked synchronization of fMRI signal fluctuations. Specifically, a method called Leading Eigenvector Dynamics Analysis (LEiDA^22^), which detects recurrent modes of phase coupling in fMRI signals, has identified subsystems that closely match functional networks reported in the literature, such as the default mode (DMN), limbic, dorsal attention (DAT), ventral attention (VAT), frontoparietal (FP), sensorimotor (SMT) and visual (VIS) networks.^23^

LEiDA can detect brain activation patterns with high temporal precision, unlike correlation-based measures of functional connectivity. At each time point, the phase relationships between N brain regions are captured by a single vector of size 1 × N, effectively circumventing many limitations of alternative methods such as the sliding-window approach, which can amplify spurious variations and lacks consensus on optimal window parameters, particularly in infant data.^24^ LEiDA has revealed functional brain states that strongly overlap with canonical “resting-state networks” or “intrinsic connectivity networks” in adults.^19,23,25^ To our knowledge, LEiDA has also been applied to infant fMRI data twice^17,26^ and to early adolescent data once,^27^ revealing meaningful networks and suggesting that these patterns can be reliably detected across human developmental stages. Specifically, in adults, the occurrence of dynamic functional networks characterized with LEiDA has been linked to cognitive performance,^22^ depressive history,^28^ effects of psychoactive drugs,^23^ and emotional reward task scores,^29^ among others. In the neonatal context, recent evidence indicates that, at birth, the human brain already exhibits a dynamic connectivity landscape that is sensitive to age at scan.^26^ Moreover, preterm-born infants show atypical dynamic connectivity patterns compared with term-born peers, which are associated with social, sensory processing, and repetitive behaviors at 18 months of age.^26^ In light of this evidence, while it is increasingly recognized that maternal health conditions during pregnancy can influence infant developmental trajectories, the dynamic properties of the neonatal brain exposed to these prenatal influences remain unknown. Specifically, it is unclear how maternal well-being, encompassing both physical and mental health, may affect dFC in neonates.^30^ While there are studies exploring maternal BMI and mental health separately in relation to early brain development, few have examined both factors together, despite their common interconnection.

In the current study, we analyzed rs-fMRI data from 437 mother-infant dyads, stemming from the developing Human Connectome Project (dHCP), applying the LEiDA method.^22^ Based on our previous study,^17^ we hypothesized that maternal BMI and depressive symptoms would be associated with the dynamics of these networks in the offspring. Our primary goal was to test the specific effects of each predictor separately while accounting for relevant covariates, and to explore potential interactions between BMI and depressive symptoms. Given the exploratory nature of this study, we did not specify directional hypotheses; rather, our analyses were designed to be hypothesis-generating, aimed at identifying potential associations that could inform future research.

## Methods

We used preprocessed data released as part of the dHCP data release 3.0, and the methods are previously described in detail.^26^ We have kept the methods description similar to this prior report for consistency.^26^

### Participants

The infants were enrolled in the dHCP study between March 2015 and March 2021. Ethical approval was granted by the National Research Ethics Committee (14/LO/1169) and written informed consent was obtained from the parents of all participants before data collection began. Participants were invited to undergo fetal MRI scan, neonatal MRI scan, or both. This study focuses on data from neonatal functional magnetic resonance imaging (fMRI), which, at the time of analysis, included data from *n* = 714 scans. Participants were excluded for the following reasons: repeat scans, lack of completed EPDS, non-singleton pregnancies, missing or incomplete fMRI data, failure to pass the dHCP image quality control (https://biomedia.github.io/dHCP-releasenotes/qc.html), gestational age (GA) at birth below 32 weeks, infant’s postmenstrual age (PMA) at scan below 37 weeks, major incidental findings identified by a pediatric neuroradiologist (e.g., arterial ischemic infarcts, brain size <1st centile), failed visual image quality control, and the absence of a successful T2-weighted scan. The final sample for the MRI analysis consisted of *n* = 437 mother-infant dyads. For each mother–infant dyad, sociodemographic characteristics and medical history were collected at enrollment through parent-report questionnaires and review of medical records (Table 1). Additional maternal variables, including mental health history and use of alcohol, tobacco, and medications during pregnancy, are presented in Table S1. Figure S1 shows EPDS distribution in mothers with and without a history of poor mental health.

**Table 1:** Descriptive statistics and frequencies for key maternal and infant variables.

Variable	*N*	Mean	SD	Median	Min	Max
GA at birth	437	39.63	1.84	40.00	32.14	42.71
PMA at scan	437	41.36	1.69	41.43	37.00	44.71
Head Circumference at scan	437	33.77	7.20	35.00	33.77	39.50
Infant Sex [female count] (%)	437	201 (46.0%)	–	–	–	–
Motion during scan	437	53.61	67.4	24	0	365
Maternal age	437	33.78	4.66	34.00	17.00	46.00
Maternal BMI	437	24.26	4.60	24.26	16.87	43.55
Maternal gestational diabetes [yes] (%)	437	23 (5.3%)	–	–	–	–
Maternal education	427	23.33	4.45	23.00	14.00	41.00
Maternal EPDS	437	5.57	4.30	5.00	0.00	28.00

Maternal education is represented as the mother’s age at the time she last attended continuous full-time education. This measure is an indirect proxy for attainment, as the same age may reflect different qualification levels.

*GA at birth* gestational age, *PMA at scan* postmenstrual age, *Motion during scan* number of outlier volumes based on DVARS interquantile range, Maternal *BMI* maternal pre-pregnancy body mass index, Maternal *EPDS* maternal perinatal depressive symptoms.

### MR image acquisition

The MR imaging was carried out using a 3T Philips Achieva scanner running modified Release 3.2.2 software. The length of the fMRI acquisition was 15 min and was collected with parameters: TE/TR = 38/392 ms, 2300 volumes, with an acquired spatial resolution of 2.15 mm isotropic voxels.^31^ To overcome challenges involved with non-sedated brain imaging of neonates, Neonatal Brain Imaging System (NBIS) was developed, consisting of a dedicated 32-channel array coil and a positioning device.^31^ The use of NBIS alongside advanced techniques for minimizing scan repeats and disturbance to the sleeping neonate has been demonstrated to improve signal-to-noise ratio 2.4 times compared to the use of an adult coil and ensure a 90% completion rate of the scan protocol.^32^

### Functional data preprocessing

The fMRI data were preprocessed using a neonatal imaging pipeline specifically optimized for the dHCP, as described in Fitzgibbon et al.^33^ Briefly, each subject underwent corrections for susceptibility dynamic distortion and both intra- and intervolume motion using a customized pipeline that included slice-to-volume and rigid-body registration.^34–37^ To mitigate signal artifacts caused by head motion, cardiorespiratory fluctuations, and multiband acquisition,^38^ 24 extended rigid-body motion parameters were regressed along with noise components identified through single-subject ICA using the FSL FIX tool (Oxford Centre for Functional Magnetic Resonance Imaging of the Brain’s Software Library, version 5.0). The denoised data were aligned to T2-weighted native space via boundary-based registration^39^ and then non-linearly registered to a standard space using a weekly template from the dHCP volumetric atlas,^40^ employing a diffeomorphic multimodal (T1/T2) registration method.^41^ We defined the timeseries for fMRI data for each participant, using the same data and procedures as in previous research.^26^ Each subject’s T2-weighted volume was parcellated into 90 cortical and subcortical regions according to the AAL (i.e., Automated Anatomical Labeling) atlas,^42^ adapted for the neonatal brain,^43^ and then manually corrected using the dHCP high-resolution template.^40^ We mapped the AAL atlas into each subject’s native space through non-linear registration utilizing a diffeomorphic symmetric normalization method (SyN),^44^ applying it to T2-weighted images and previous segmentations. Gray matter segmentation and parcels were then transformed from T2 native space to fMRI space using a boundary-based linear registration method within the functional dHCP processing pipeline.^33^ Finally, average timeseries were extracted for each of the 90 cortical and subcortical AAL areas. We employed FSL’s ’fsl_motion_outliers’ to detect volumes where DVARS (the root mean square intensity difference between consecutive volumes) exceeded 1.5 times the interquartile range (IQR) above the 75th percentile, following motion and distortion correction.^45^ These volumes were flagged as motion outliers. The count of these motion-outlier volumes for each subject was documented and later used as a covariate in statistical analyses.

### Temporal alterations in brain networks

We ran all the modeling in MATLAB 2022. Before running LEiDA, we first obtained the average fMRI signals from 90 cortical and subcortical areas defined by the UNC neonate AAL atlas using FSL fslmeants.^46^ The time courses were bandpass filtered (0.02–0.10 Hz), the analytic phase was obtained using the Hilbert transform, and the leading eigenvectors of the phase coherence matrices were calculated at each time point.^22^ The eigenvectors were then clustered via K-means clustering using cosine distance with 200 replicates. For this exploratory study, the number of clusters (K) was varied between 2 and 10, based on previous studies suggesting that the optimal number of intrinsic networks is typically between 3 and 8.^17,23,25,27^ Clustering yields K states of brain activity, each representing a recurrent pattern of functional connectivity that can be characterized using measures derived from LEiDA.^22^ Specifically, we considered: (i) probability of occurrence, the proportion of time spent in each state, reflecting its dominance; (ii) lifetime, the mean duration a state persists once entered, indexing stability; and (iii) transitions, the likelihood of switching between states, reflecting network flexibility. In the present study, we examined associations between maternal pre-pregnancy BMI and perinatal depressive symptoms with the probability of occurrence and transitions of the identified brain states, as these measures are particularly informative about state dominance (i.e., probability of occurrence) and dynamic shifts (i.e., transitions). For completeness, lifetimes and full transition probability matrices are reported in the Supplementary Material (Figs. S3 and S4).

### Maternal depressive symptoms

Maternal depressive symptoms were assessed using the EPDS^47^ at each study visit. The EPDS is a 10-item questionnaire with a total score ranging from 0 to 30. Scores from the EPDS were included in the analysis if they were obtained during pregnancy (e.g., during a fetal scan visit) or in the early postnatal period (e.g., at the first neonatal scan), as these instances are indicative of mood during the prenatal and early postnatal phases. As the EPDS only asks about symptoms over the last 7 days, in cases where participants completed more than one EPDS questionnaire, the highest score was chosen. EPDS scores in our sample were available as follows: *n* = 60 only prenatal timepoints, *n* = 362 only postnatal timepoint, *n* = 15 both prenatal and postnatal timepoints. For prenatal assessments, the median GA was 30.4 weeks, with a range from 22.5 to 41.6 weeks. For postnatal assessment, the median PMA was 41.2 weeks, with a range from 33.1 to 44.7 weeks.

### Statistical analysis

Analyses were performed using the statistical software R.^48^ First, we tested the associations between LEiDA-derived network probabilities of occurrence (extracted from MATLAB into conventional tabular data) and maternal BMI during pregnancy, as well as maternal perinatal depressive symptoms, using General Linear Models (GLMs). We then performed another series of GLMs to test the associations between LEiDA-derived network transitions, maternal BMI during pregnancy, and perinatal depressive symptoms. Covariates were chosen based on previous literature and include infants’ PMA at scan, sex, motion during scan, maternal age and gestational diabetes. For completeness, in the supplementary material, we report the results of the same models regarding the lifetime of brain networks (Fig. S11). Bivariate correlations between covariates and variables of interest are also reported in Supplementary Material, Section 3 (Figs. S5, S6, S7). To account for multiple comparisons, we applied the Bonferroni correction to the statistical tests based on the number of clusters, that is, for each independent hypothesis tested within each clustering solution. We reported uncorrected and Bonferroni corrected *p* values. Finally, we performed a sensitivity analysis excluding preterm infants (*N* = 33) and then including GA at birth, maternal medication use (Table S1) and smoking (*N* = 11) during pregnancy as covariates to assess their potential influence on results.

## Results

### Neonatal brain states

**Fig. 1:**
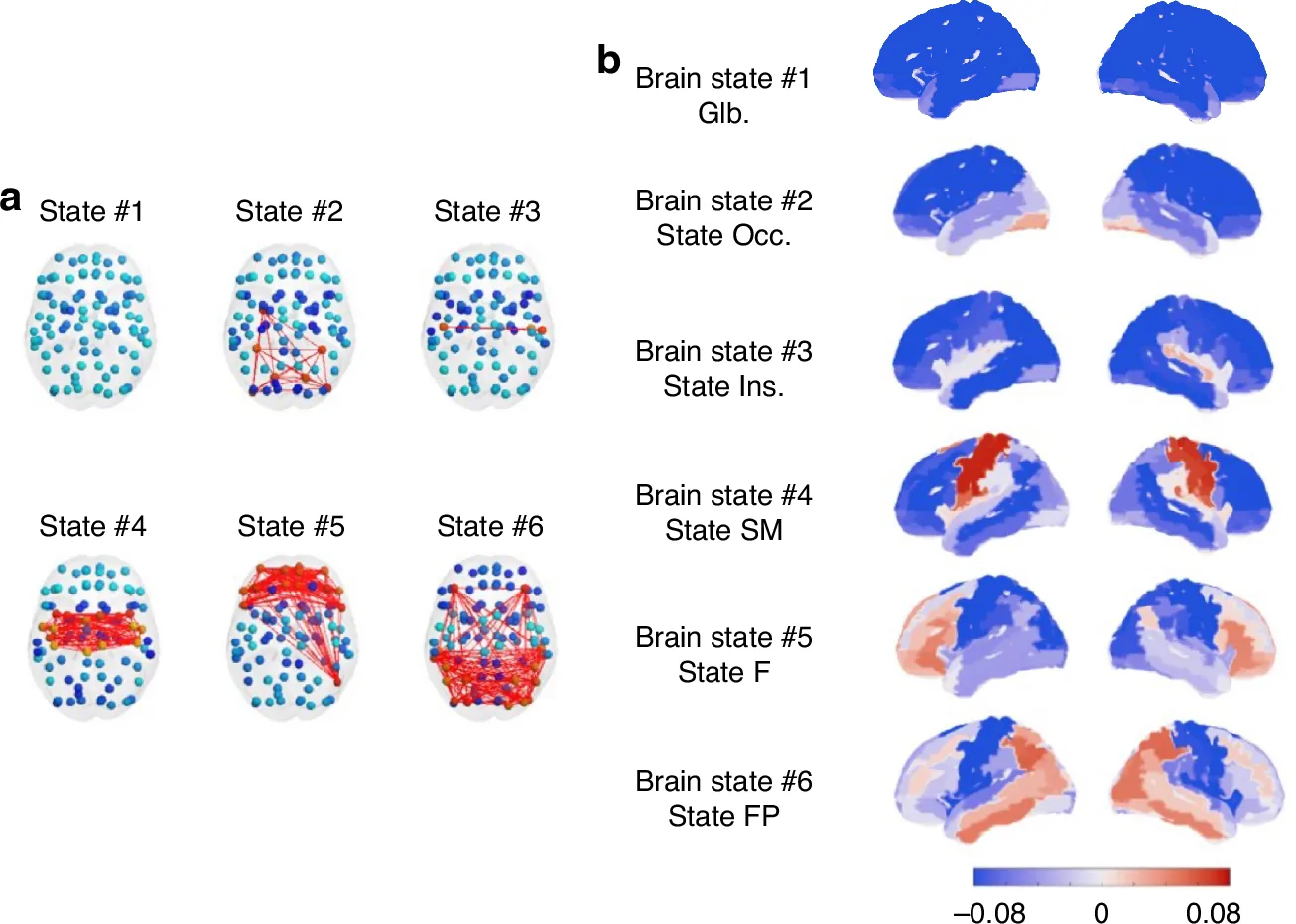
Neonatal brain states identified with LEiDA (K = 6). Brain states are visualised in **a** showing red links between the brain areas that temporarily align their phase to another direction with respect to other brain areas, and **b** cortical representation coloring these areas in red, with the scale representing the cosine of their phase difference such that cos(0) = 1 represents in-phase synchrony, while -1 (blue) represents a phase difference of 180°. Glb global, Occ occipital, Ins insula, SM sensorimotor, F frontal, FP frontoparietal.

Consistent with prior studies on infants, we set the K-means clustering solution with *K* = 6 distinct brain states for the main analyses (Fig. 1).^17,26^ In line with a previous study in infants that used an overlapping sample,^26^ we identified a brain state of global signal coherence #1 (Glb. State), and five brain states that exhibited spatial patterns that resemble neonatal resting state networks^49^: brain state #2 (Occipital State) was characterized by synchronous activity in the occipital cortex, brain state #3 (Insular State) encompassed the bilateral insulae, brain state #4 (SM State) showed synchrony in the bilateral sensorimotor cortices, brain state #5 (F State) in frontal areas and brain state #6 in the frontal cortex, angular gyrus, and posterior cingulate gyrus, which we refer to as the frontoparietal state (FP State) for simplicity. The repertoire of states obtained with varying the number of clusters between *K* = 2 to *K* = 10 is reported in Supplementary Material (Fig. S2).

### Association between maternal prenatal BMI and perinatal EPDS with brain state probability of occurence

**Fig. 2:**
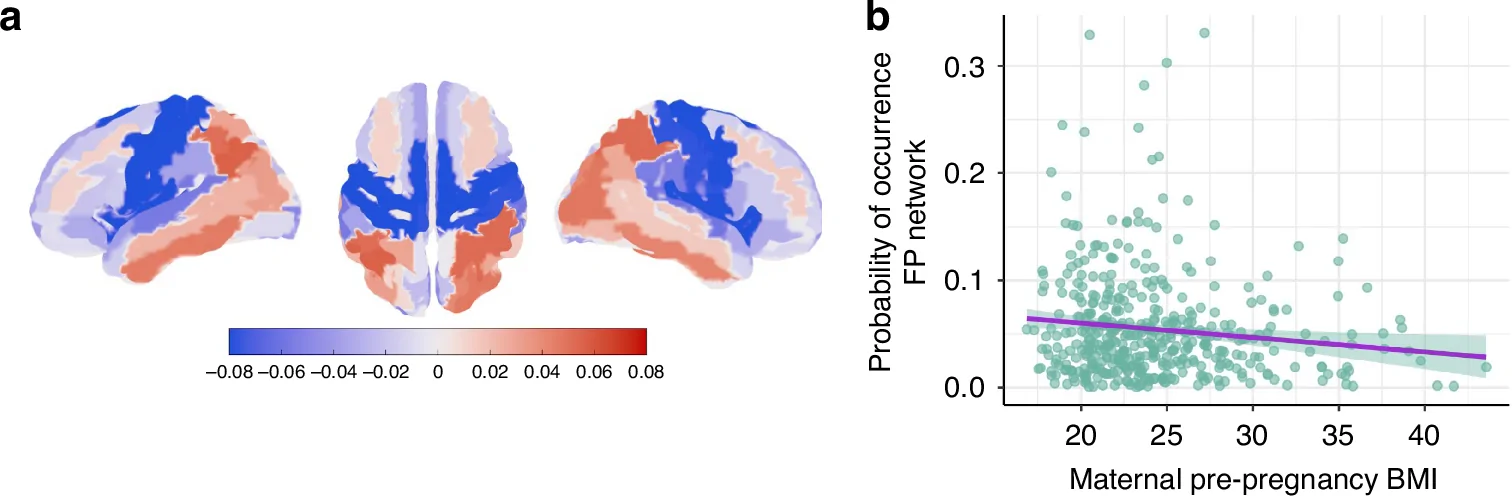
Maternal pre-pregnancy BMI associates negatively with the probability of occurrence of a frontoparietal brain network in neonates. **a** Visualization of the network, and **b** scatter plot of the association. FP frontoparietal, BMI body mass index.

Maternal pre-pregnancy BMI was negatively associated with the probability of occurrence of the FP functional network (i.e., brain state #6), which encompasses the superior frontal, superior parietal, and temporal brain regions (*β* = −0.129, *p* = 0.006) (Fig. 2). While this association had a modest effect size, it remained robust after adjusting for covariates (i.e., infant’s PMA at scan, sex, motion during scan, maternal age, and gestational diabetes, Table 2) and survived Bonferroni correction (*p*_Bonf_ = 0.036). Notably, as the frontoparietal state (i.e., state #6) shows a consistent spatial configuration across higher clustering solutions (*K* = 7, 8, 10), we tested the association between pre-pregnancy BMI and its probability of occurrence also when *K* was set at 7, 8, and 10. We observed that this association remained consistently negative and statistically significant (Tables S4, S5, S6). The uncorrected and corrected *p* values for each brain state were as follows: brain state #7 for *K* = 7 (*β* = −0.132, *p* = 0.006, *p*_Bonf_ = 0.041); brain state #8 for *K* = 8 (*β* = −0.130, *p* = 0.006, *p*_Bonf_ = 0.048); and brain state #10 for *K* = 10 (*β* = −0.133, *p* = 0.005, *p*_Bonf_ = 0.037). Since our sample included preterm infants, we conducted a sensitivity analysis to test the robustness of our findings. The association between maternal pre-pregnancy BMI and the probability of occurrence of the FP network remained consistent when excluding N = 33 preterm infants (*β* = −0.14, *p* = 0.006, *p*_Bonf_ = 0.045; Table S7) and when including GA at birth as a covariate (*β* = −0.14, *p* = 0.006, *p*_Bonf_ = 0.048; Table S8). In the Supplementary Material (Section 5), we report sensitivity analyses including other relevant variables such as maternal use of medications (Table S9) and smoking during pregnancy (*N* = 11; Table S10), none of which altered the results. In the Supplementary Material we provided more details on the FP network (Fig. S8)—including specific brain regions based on the AAL atlas - the associations between maternal prepregnancy BMI and the probability of the occurrence of all brain states when *K* = 6 (Fig. S9), and the relationship between maternal pre-pregnancy BMI and the lifetime of brain states (Fig. S12).

**Table 2:** Model results.

Term	Estimate	SE	*p*	p_Bonf
Maternal EPDS	0.0005	0.0005	0.370	1.00
Maternal BMI	−0.0014	0.0005	0.006	0.036
Maternal age	0.0001	0.0005	0.942	1.00
Gestational diabetes	−0.003	0.0104	0.738	1.00
Infant sex	0.0094	0.0046	0.042	0.294
Infant PMA at scan (weeks)	−0.0026	0.0014	0.064	0.449
Motion	0.0002	0.0003	<0.001	<0.001

The table includes standardized beta coefficients (Estimate), standard errors (SE), and *p* values. Adjusted *R*² for the model is 0.10. Maternal EPDS; Maternal perinatal depressive symptoms, Maternal *BMI* Maternal Body Mass Index, Infant PMA at scan; postmenstrual age at scan, Motion; infants motion during scan.

Maternal perinatal depressive symptoms were not associated with the occurrence of any of the functional networks examined. Although beyond the primary scope of this study, we explored whether maternal pre-pregnancy BMI and depressive symptoms interact in shaping FP network occurrence. However, these results did not survive correction for multiple comparisons and are reported in the Supplementary Material (Fig. S10, Table S2; Fig. S11, Table S3).

### Association between maternal prenatal BMI and perinatal EPDS with brain state transitions

**Fig. 3:**
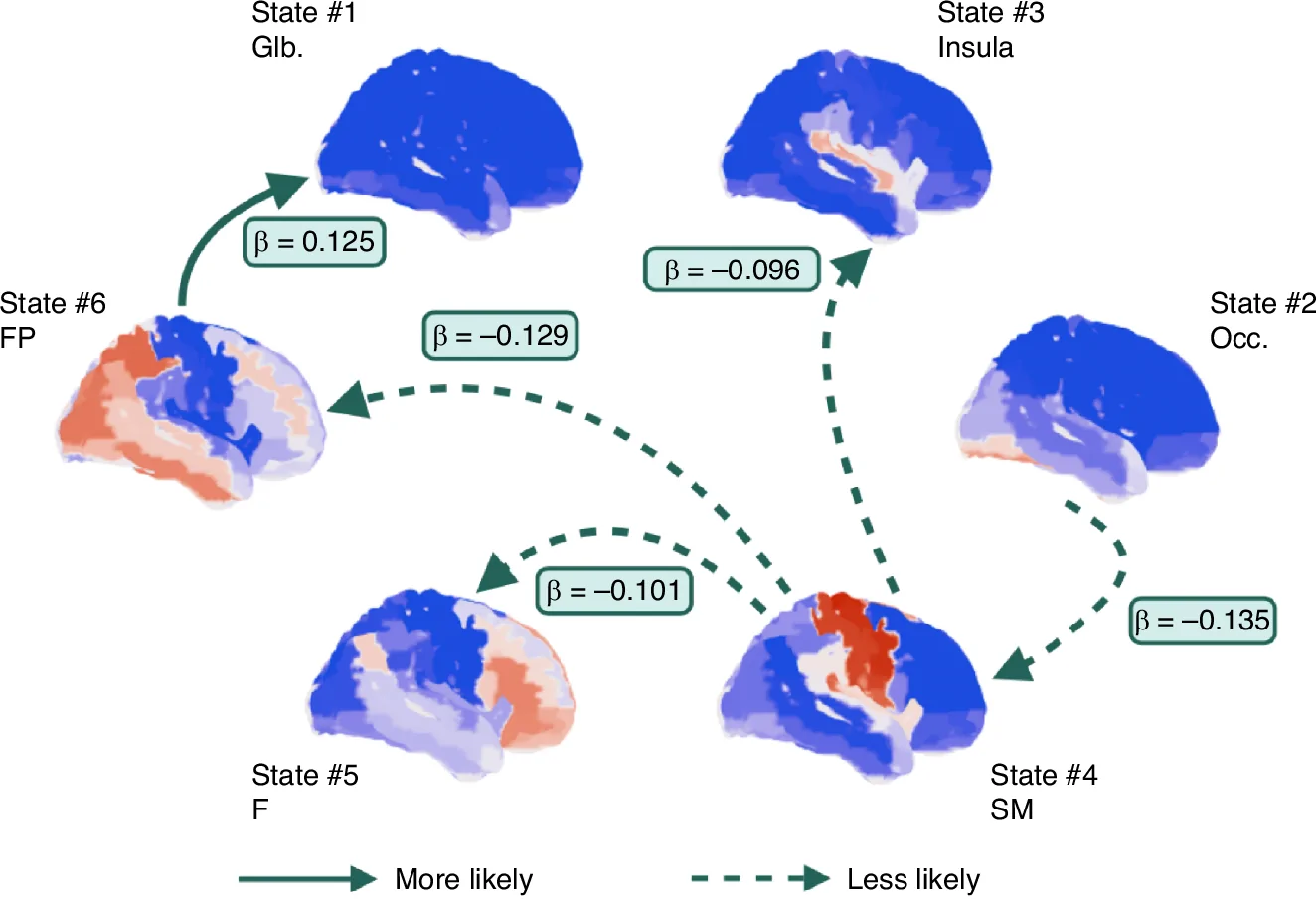
Probability of transition and maternal pre-pregnancy BMI (*K* = 6). The probability of transition from state #2 to #4, from #4 to #3, from #4 to #5 and from #4 to #6 is negatively predicted by maternal pre-pregnancy BMI. The probability of transition from state #6 to global state A is positively predicted by maternal pre-pregnancy BMI. Glb global, Occ occipital, SM sensorimotor, F frontal, FP frontoparietal, BMI body mass index.

Figure 3 shows the associations between maternal pre-pregnancy BMI and brain state transitions. Higher maternal BMI was significantly associated with a reduced likelihood of transitioning from the occipital state #2 to the sensorimotor state #4 (*β* = −0.135, *p* = 0.006, *p*_Bonf_ = 0.048). Additional associations were observed but did not survive correction for multiple comparisons: higher BMI was related to a lower probability of transitioning from the sensorimotor state #4 to the frontoparietal state #6 (*β* = −0.129, *p* = 0.008, *p*_Bonf_ = 0.064), from the sensorimotor state #4 to insular state #3 (*β* = −0.096, *p* = 0.045, *p*_Bonf_ = 0.384), and from the sensorimotor state #4 to the frontal state #5 (*β* = −0.101, *p* = 0.036, *p*_Bonf_ = 0.304). In contrast, BMI was associated with an increased likelihood of transitioning from the frontoparietal state #6 to the global state A (*β* = 0.125, *p* = 0.011, *p*_Bonf_ = 0.088).

EPDS scores, were not associated with transitions of any of the functional networks examined.

## Discussion

### Dynamic connectivity in the neonate brain

Our study describes that the human brain establishes a well-organized dynamic connectivity landscape in the brain soon after birth. Consistent with earlier research using both fMRI^50,51^ and EEG,^52^ we found that newborns spend a significant amount of time in a state of global phase synchrony. Whole-brain synchrony state 1 exhibited the greatest fractional occupancy, indicating a dominant pattern of whole-brain synchronization. The remaining five states strongly overlap with functional connectivity patterns previously identified in pediatric populations.^53–55^ Specifically, the second and fourth states were characterized by a strong involvement of auditory, sensorimotor, and visual regions, aligning with evidence that sensory networks are among the first to emerge and are robustly expressed in infancy.^45,56,57^ In contrast, the third state was dominated by regions of the salience network,^58^ notably the insula and anterior cingulate, which are detectable in infancy but undergo substantial refinement over development.^59^ Finally, the fifth and sixth states involved dorsal attention and frontoparietal control networks,^58^ which mature later in childhood, consistent with their lower probability of occurrence in our data. This consistency reinforces the findings of studies on dFC in newborns and underscores the importance of large-scale brain activity for early development.^60,61^ Such activity likely supports the emergence of widespread cortical networks and long-range connections that continue to mature throughout the first postnatal year, providing a foundation for sensory, motor, and higher-order cognitive functions.

### Intergenerational implications of maternal overweight

We observed a negative association between the probability of occurrence of a functional network encompassing the superior frontal, superior parietal, and temporal regions and maternal pre-pregnancy BMI. This aligns with prior evidence that maternal metabolic health influences fetal brain development^4–6,8,9,62–64^ and highlights the particular sensitivity to this effect of frontal regions. Indeed, previous studies have linked different properties of infants’ frontal regions to maternal BMI, reporting both reduced cortical thickness^62^ and altered static functional connectivity.^5,6^ Our dynamic modeling approach extends these findings by showing that higher maternal BMI is associated with reduced stability of fronto-parieto-temporal states. Since the FP network becomes increasingly prominent with postnatal age,^26^ the reduced probability of occurrence observed here suggests delayed maturation of this system. Moreover, several regions within the FP network overlap with the anterior Default Mode Network (DMN),^65^ which undergoes substantial reorganization in early life through progressive recruitment of frontal areas. The reduced occurrence of these regions in association with maternal BMI therefore reinforces the notion of delayed network development. These alterations also echo reports of atypical DMN patterns in overweight and obese adults,^66–68^ which have been linked to poorer cognitive outcomes later in life.^67,68^ Another indicator of the destabilizing effect of maternal weight on infant brain development is the finding that higher maternal pre-pregnancy BMI is associated with a reduced likelihood of transitions from the occipital to the sensorimotor state and from the sensorimotor to the frontal and fronto-parietal states. Typically, transitions from occipital to sensorimotor states support visuomotor development and sensory integration,^69^ whereas transitions from sensorimotor to fronto-parietal states reflect the emerging maturation of higher-order networks. This developmental trajectory coincides with substantial interneuron migration into frontal and parietal regions,^70^ in contrast to the relatively mature sensorimotor systems.^71^ Disruptions in these early transition patterns may compromise the integration of visuomotor and higher-order functions, helping to explain why children born to overweight or obese mothers often show deficits in motor coordination^72^ and suboptimal visuomotor skills.^73,74^ Critically, these very early alterations in network dynamics across occipital, sensorimotor, and fronto-parietal networks may set the stage for later atypical connectivity patterns in multiple brain systems. In adults, such atypical patterns have been linked to impairments in attention, working memory, executive and inhibitory control, and broader cognitive control dysfunction—a phenotype commonly observed in overweight and obese individuals that can contribute to dysregulated eating and energy imbalance.^75–77^Taken together, the present findings suggest that the brain dynamics of neonates born to mothers with higher BMI already mirror patterns associated with later behavioral and cognitive challenges. Although we did not assess mechanisms directly, hormonal and inflammatory changes associated with maternal obesity have been proposed as potential contributors to these intergenerational effects, highlighting the impact of maternal metabolic health on early neurodevelopment.

### Limitations and future directions

While this study provides valuable insights, some limitations must be considered. First, our study is cross-sectional and thus does not allow for causal inference. Second, the scale of depressive symptoms is unable to capture the potential mediating biological or physiological mechanisms. Furthermore, EPDS only captures symptoms in the last 7 days; this narrow window may not fully reflect mental health throughout the perinatal period, as scores could be influenced by transient fluctuations. In addition, very few participants with high EPDS scores could have affected associations between maternal perinatal depressive symptoms and neonatal brain connectivity. Third, although BMI is a well-established measure of weight status, it does not directly measure metabolic health, and we were unable to take into account changes in weight during pregnancy. It should also be mentioned that BMI does not distinguish between fat and muscle mass, which means that people with high muscle mass may have a higher BMI despite having low body fat.^78^ Fourth, motion during scanning was a significant covariate in our analyses, and although we adjusted for it statistically, its influence represents a potential limitation. Finally, although the dHCP includes longitudinal developmental outcome data, linking neonatal brain dynamics to later behavioral or cognitive trajectories was beyond the scope of the present study and should be addressed in future work. Future research incorporating longitudinal designs and objective measures of biological processes, such as hormonal changes, inflammatory markers, and genetic factors, could provide deeper insight into the underlying mechanisms linking maternal mental health with early brain development in offspring.

## Supplementary information

Supplementary information

## Data Availability

The AAL-UNC atlas adapted to the dHCP template space, and pre-processed BOLD timeseries data generated in this study have been deposited in the Zenodo database under accession code https://doi.org/10.5281/zenodo.7053984. The fMRI datasets and clinical data are available under restricted access as per dHCP data release conditions, access can be obtained via https://data.developingconnectome.org. The corresponding author is responsible for submitting a competing interests statement on behalf of all authors of the paper. This statement must be included in the submitted article file.
