# Supplementary material for: Investigating the influence of maternal prenatal BMI and perinatal depressive symptoms on neonatal brain network dynamics
Source: Pediatr Res. 2026 Jan 15;100(2):757–64. doi: 10.1038/s41390-025-04726-2 (PMC13638477; doi:10.1038/s41390-025-04726-2)
Supplement: Supplementary file 1 [file 41390_2025_4726_MOESM1_ESM.pdf]

# Investigating the Influence of Maternal Prenatal BMI and Perinatal Depressive Symptoms on Neonatal Brain Network Dynamics

Mariani Wigley Isabella L.C, Lautarescu Alexandra, Vartiainen Elena, Pulli Elmo P, Hashempour Niloofar, Merisaari Harri, Bano Wajiha, Luotonen Silja, Jolly Ashmeet, Suuronen Ilkka, Karlsson Linnea, Karlsson Hasse, Cabral Joana, Kringelbach ML, Batalle Dafnis, Edwards A. David and Tuulari Jetro J

***Supplementary Material***

Contents:

1. Comprehensive maternal information during pregnancy

2. Neonates Brain
   2.1 Pyramid LEiDA network plot of neonates' functional connectivity (FC) states
   2.2 Lifetimes
   2.3 Transitions

3. Correlation matrix between key variables
   3.1 Probability of occurrence
   3.2 Lifetime
   3.3 Transitions

4. Maternal prenatal BMI
   4.1 Neonates' frontoparietal network
   4.2 Maternal prenatal BMI and brain state probability of occurrence (k = 6)
   4.3 Exploring the interaction between maternal prenatal BMI and Perinatal depressive symptoms in predicting brain state probability of occurrence (k = 6)
   4.4 Maternal prenatal BMI and the probability of occurrence of the frontoparietal network
   4.5 Maternal prenatal BMI and Lifetime

5. Sensitivity

## Abstract

Background: Elevated pre-pregnancy body mass index (BMI) and perinatal depressive symptoms have been linked to neonatal alterations in brain structure and function. This study examined associations between neonatal functional brain dynamics, maternal BMI, and perinatal depressive symptoms measured by the Edinburgh Postnatal Depression Scale (EPDS) in a community-based, largely low-risk cohort.

Methods: funcitonal MRI and Leading Eigenvector Analysis (LEiDA) were applied in a neonatal cohort ($N = 437$; 236 males; mean gestational age 39.6 weeks) from the developing Human Connectome Project. We assessed whether neonatal brain-state probabilities related to maternal BMI and EPDS scores ($M = 5.6$, $SD = 4.3$), testing main effects and, separately, their interaction. The sample included 291 healthy-weight (BMI < 25), 98 overweight (25 BMI < 30), and 48 obese (BMI 30) mothers.

Results: EPDS scores were low in this cohort and did not demonstrate associations with brain states or a significant BMI x EPDS interaction. Higher maternal pre-pregnancy BMI was negatively associated with the stability of a functional network encompassing superior frontal, superior parietal, and temporal regions (ß = -0.129, $p = 0.006$).

Conclusion: As this network is normally recruited more with age, reduced stability suggests slowed maturation of fronto-parieto-temporal systems and may signal early risk for later behavioral challenges.

## 1. **Comprehensive maternal information during pregnancy**

Table S1 summarizes key maternal variables, including history of poor mental health, anxiety and depression, and the use of alcohol, smoking, and medications during pregnancy. As part of the dHCP maternal questionnaire pack, participants were asked: “Have you ever been treated for a mental health problem?” (Yes/No). If they answered “No,” no further questions were administered. If they answered “Yes,” three follow-up Yes/No questions were posed: “Have you ever been under psychiatric services?”, “Have you ever been admitted to hospital for a mental health problem?”, and “Do you have a history of ADHD, bipolar disorder, depression, autism, or schizophrenia?” To obtain a more comprehensive record of maternal mental health, where availbale, information was collated from multiple sources, including maternal self-report, electronic and hand-held maternity notes, and records from the South London and Maudsley NHS Foundation Trust. Using these sources, we derived a composite variable, History of poor mental health. This was coded “Yes” if mothers reported treatment for a mental health problem in the dHCP questionnaire or if corroborating evidence was found in any of the other data sources; otherwise, it was coded “No.”

The distribution of EPDS scores in mothers with and without a history of poor mental health is shown in Figure S1.

Regarding maternal medication use during pregnancy, medications reported across the four study visits were categorized into five groups. The first group comprised over-the-counter drugs, vitamins, and supplements, including commonly used products such as paracetamol, aspirin, folic acid, iron, calcium, multivitamins, Pregnacare, omega-3, ferrous sulphate, and Gaviscon. The second group consisted of corticosteroids, including both systemic and inhaled formulations such as prednisolone, budesonide, beclomethasone, and Becotide. The third group included antidepressants, specifically selective serotonin reuptake inhibitors (SSRIs) and serotonin–norepinephrine reuptake inhibitors (SNRIs), namely sertraline and venlafaxine. A fourth group captured any other prescription or non-prescription drugs not included in the above categories. Finally, a fifth group represented participants who reported no medications, supplements, or over-the-counter products. For classification, participants were assigned the highest-priority category applicable to any reported drug, with antidepressants taking precedence over corticosteroids, and corticosteroids taking precedence over over-the-counter drugs and supplements.

| Variabile | Category | Frequency | Percentage |
|---|---|---|---|
| Maternal History poor MH | 0 | 325 | 74.4 |
| | 1 | 109 | 24.9 |
| | Missing | 3 | 0.7 |
| Maternal history of anxiety | 0 | 410 | 93.8 |
| | 1 | 24 | 5.5 |
| | Missing | 3 | 0.7 |
| Maternal history of depression | 0 | 379 | 86.7 |
| | 1 | 55 | 12.6 |
| | Missing | 3 | 0.7 |
| Alcohol | 0 | 396 | 90.6 |
| | 1 | 41 | 9.4 |
| Smoking | 0 | 426 | 97.5 |
| | 1 | 11 | 2.5 |
| Maternal medications during pregnancy | 1 | 371 | 84.9 |
| | 2 | 12 | 2.7 |
| | 3 | 5 | 1.1 |
| | 4 | 17 | 3.9 |
| | 5 | 32 | 7.3 |

***Table S1*. *Key maternal variables during pregnancy.*** *MH, mental health; 0 = no, 1 = yes, missing = missing values; Maternal medications during pregnancy, 1 = over-the-counter drugs; 2 = corticosteroids, 3 = SSRIs, 4 = other medications, 5 = no medications.*

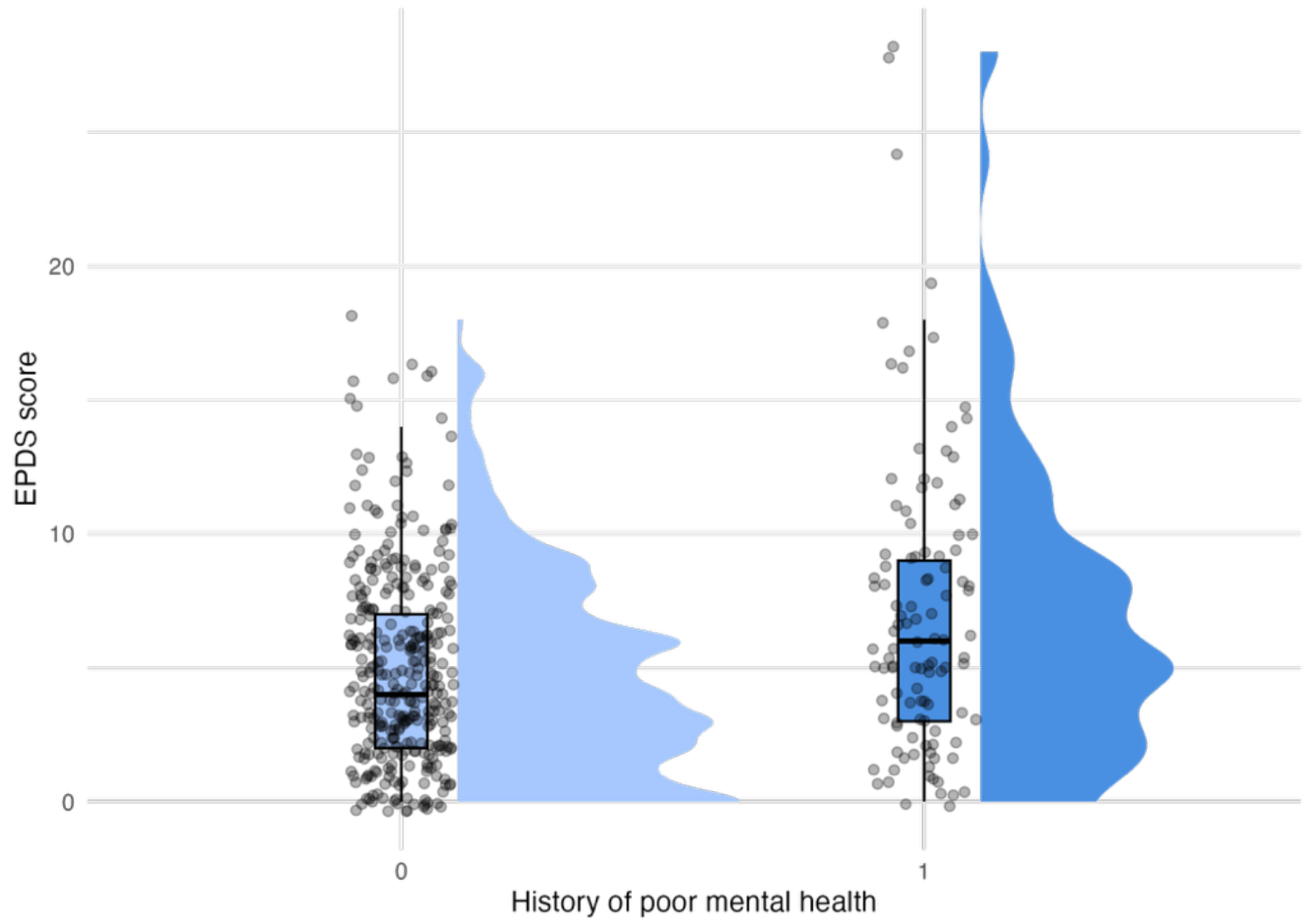


***Figure S1*. *EPDS distribution in mothers with and without a history of poor mental health*.** *Raincloud plot showing the distribution of highest perinatal EPDS scores in participants with (1) and without (0) a history of poor mental health. Jittered points represent individual data, boxplots display the median and interquartile range, and half-eye density plots show the distribution alongside each group.*

## 2. Neonatal brain states

We characterized neonatal brain states using Leading Eigenvector Dynamics Analysis (LEiDA) (Cabral et al., 2017), a data-driven approach to study dynamic functional connectivity (dFC). LEiDA extracts the leading eigenvector of phase coherence matrices at each time point, providing a low-dimensional representation of whole-brain dynamics and capturing recurrent phase-locking (PL) states. Instantaneous BOLD phases were estimated using the Hilbert transform (Boashash, 1992), yielding the analytic signal:

$$x(t) = A(t) \cos [\theta(t)]$$

where $A(t)$ is the instantaneous amplitude and $\theta(t)$ the instantaneous phase. Pairwise phase coherence between regions $n$ and $m$ at time $t$ was then computed as:

$$\text{dFC}(n, m, t) = \cos (\theta(n, t) - \theta(m, t))$$

This produces a symmetric N × N dFC matrix for each time frame $t$.

Rather than comparing full dFC matrices, LEiDA considers only the dominant connectivity pattern, represented by the leading eigenvector $V_1(t)$ of each dFC matrix. This vector captures the main phase-locking configuration and explains > 50% of variance (Cabral et al., 2017). Each $V_1(t)$ can be treated as a point in $R^N$, yielding a trajectory of whole-brain dynamics (Vohryzek et al., 2020). To identify recurrent PL states, we applied k-means clustering to the set of eigenvectors across all time points and subjects (Cabral et al., 2017; Vohryzek et al., 2020). The algorithm partitions eigenvectors into k clusters, with centroids $V_{C\alpha}$ $(\alpha=1,\ldots,k)$ representing distinct PL states $R\alpha$. Each time point is assigned to the nearest centroid, producing a discrete trajectory in state space. Although Hidden Markov Models (Vidaurre et al., 2016) can also be used, Cabral et al. (2017) showed that k-means yields comparable results and is computationally efficient.

## 2.1 Pyramid LEiDA network plot of neonates' FC states

In this section, we report neonates' brain functional states detected for increasing number of clusters K (with K = 2-10) and sorted from left to right according to probability of occurrence or also know as fractional occupancy (FO; Vohryzek et al. 2020; Alonso & Tyborowska 2023). FO is defined as the number of times when the trajectory $x(t)$ is assigned to each of the defined clusters $R\alpha$ divided by the total number of time points $T$, assuming stationarity within each scan since subjects are constantly in resting-state. It is important to note that state numbers are assigned independently within each K solution and do not indicate direct correspondence across different values of K.

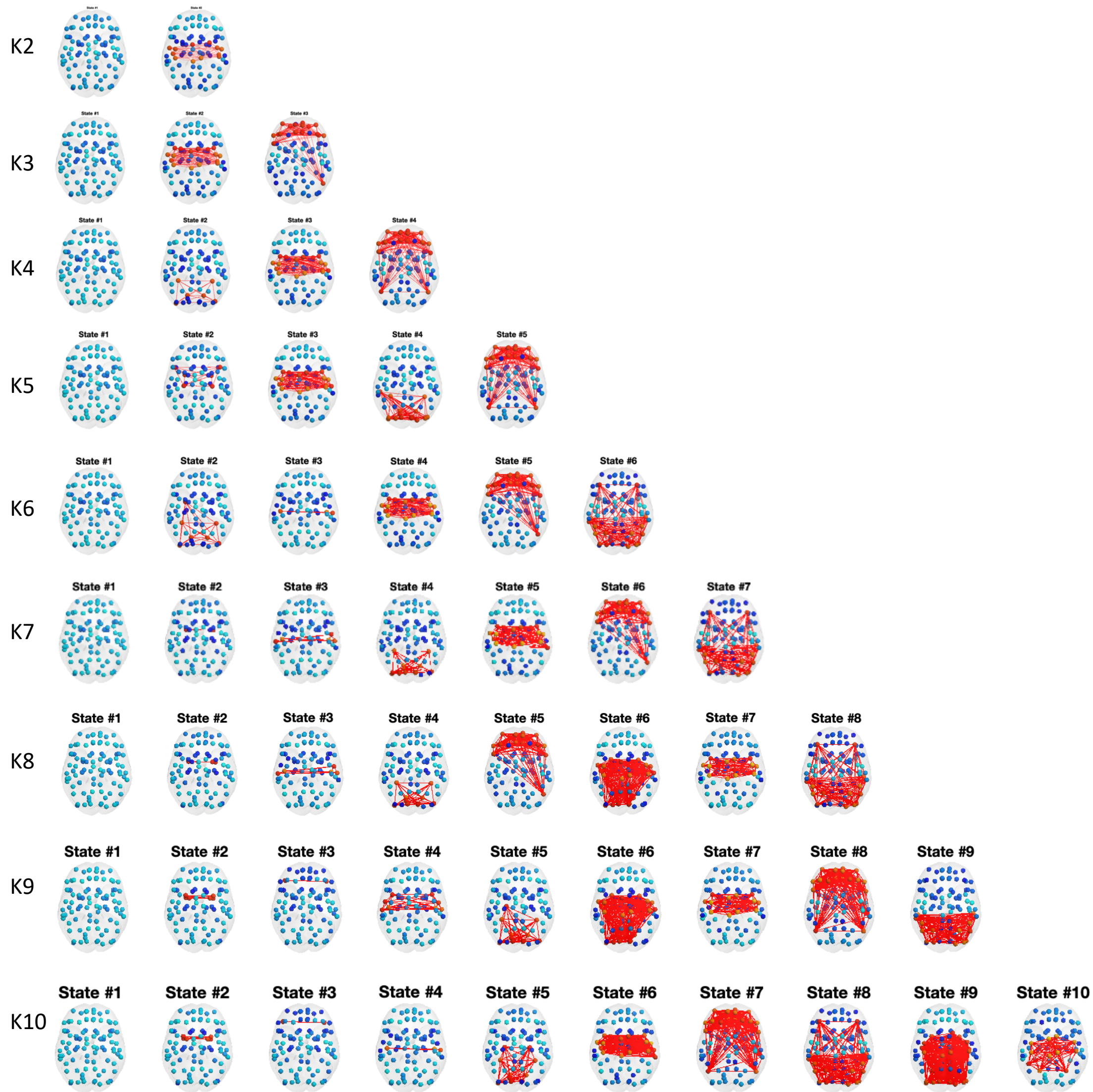


***Figure S2. Pyramid LEiDA network plot.*** *LEiDA network pyramid depicting neonates' brain functional states detected for increasing number of clusters K (with K = 2-10) and sorted from left to right according to probability of occurrence. Note that state numbers are assigned independently within each K solution and do not indicate direct correspondence across different values of K.*

### 2.2 Lifetimes

Lifetime, or Dwell Time, refers to the time spent in a functional connectivity state (Vohryzek et al. 2020; Alonso & Tyborowska 2023). It is defined as the mean of all the consecutive periods of each state, and is used to describe the average time periods when a given PL state α is being visited in each fMRI scan *S*.

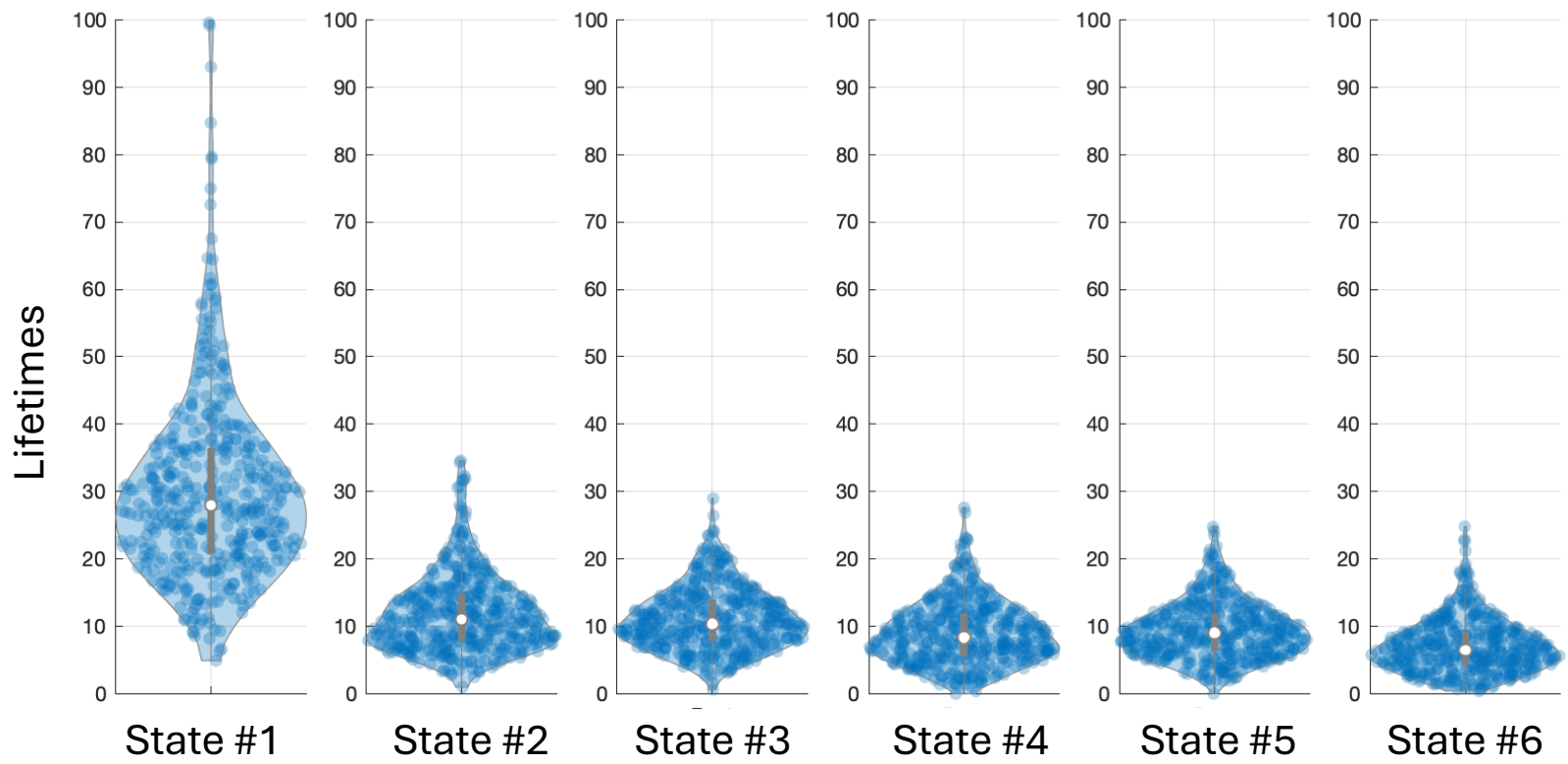


***Figure S3. Lifetimes of neonates' brain states (K = 6).*** *It represents the time spent in each functional connectivity state when K = 6.*

### 2.3 Transitions

This metric refers to the probability of transitions between different brain states. Markov Chain transition probability (Vohryzek et al. 2020; Alonso & Tyborowska 2023) matrix $W^{(S)}_{\alpha\beta}$ of each fMRI scan *S* defines the transition matrix from state *α* to state *β*. The obtained transition matrix defines a homogeneous Markov Chain, characterizing the transition between BOLD phase locking states, and is estimated separately for each scan *S*.

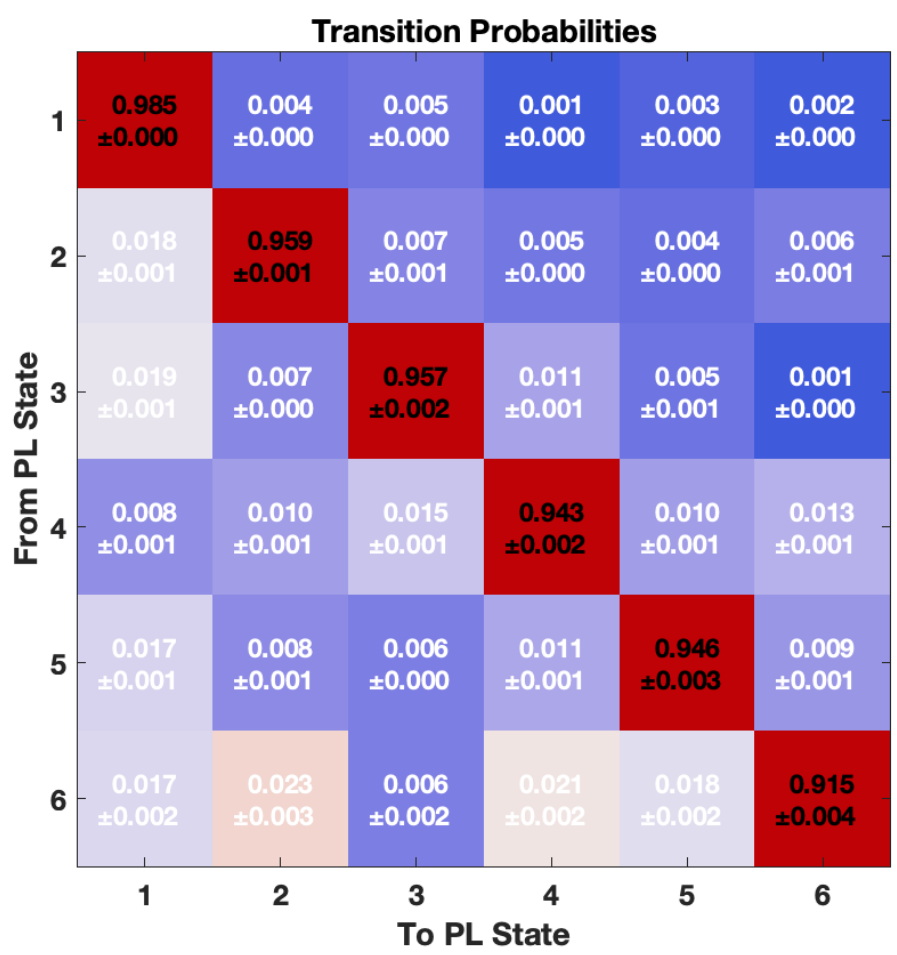


***Figure S4. Transition probabilities matrix between neonates' brain states (K = 6).*** *It indicates the probability of, being in each FC state (lines), transitioning to any of the other states (columns) and +/- standard deviations. PL: recurrent BOLD phase-locking (PL) patterns.*

## 3. Correlation matrix of key variables

Below, we present the correlation matrices computed for maternal and neonates' variables included in the model, and brain network characteristics—namely, occurrence probability, lifetime, and transition probability—derived for K = 6.

### 3.1 Probability of occurrence

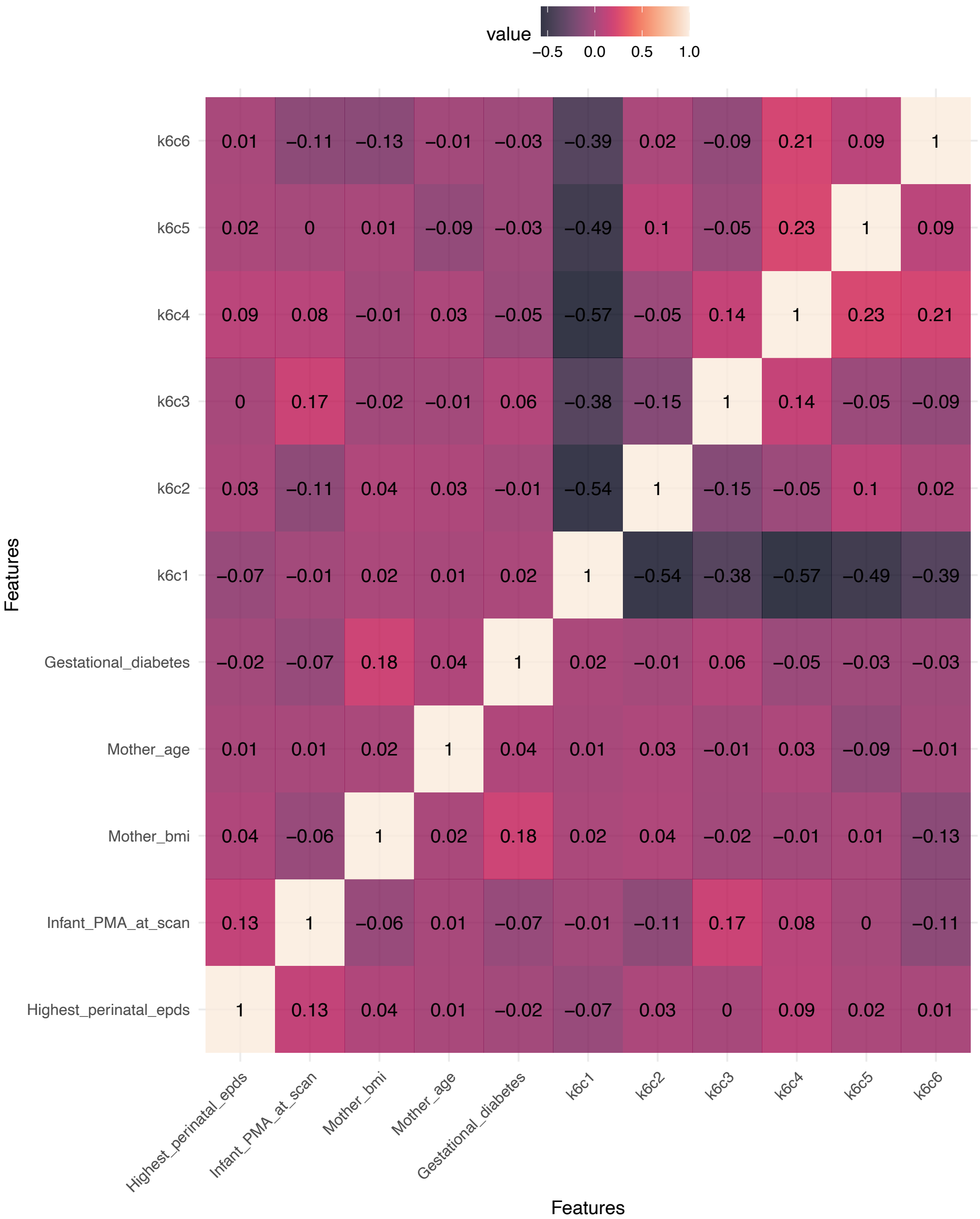


**Figure S5. Bivariate correlation coefficients between variables of interest and probability of occurrence of brain states (K = 6).** *Pearson's bivariate correlation coefficient is reported. k6c1–k6c6: probability of occurrence of brain states #1–#6 when K = 6. Mother_bmi, maternal pre-pregnancy BMI; Infant_PMA_at_scan, infant postmenstrual age at scan (in weeks); Highest_perinatal_epds, maternal perinatal depressive symptoms.*

### 3.2 Lifetime

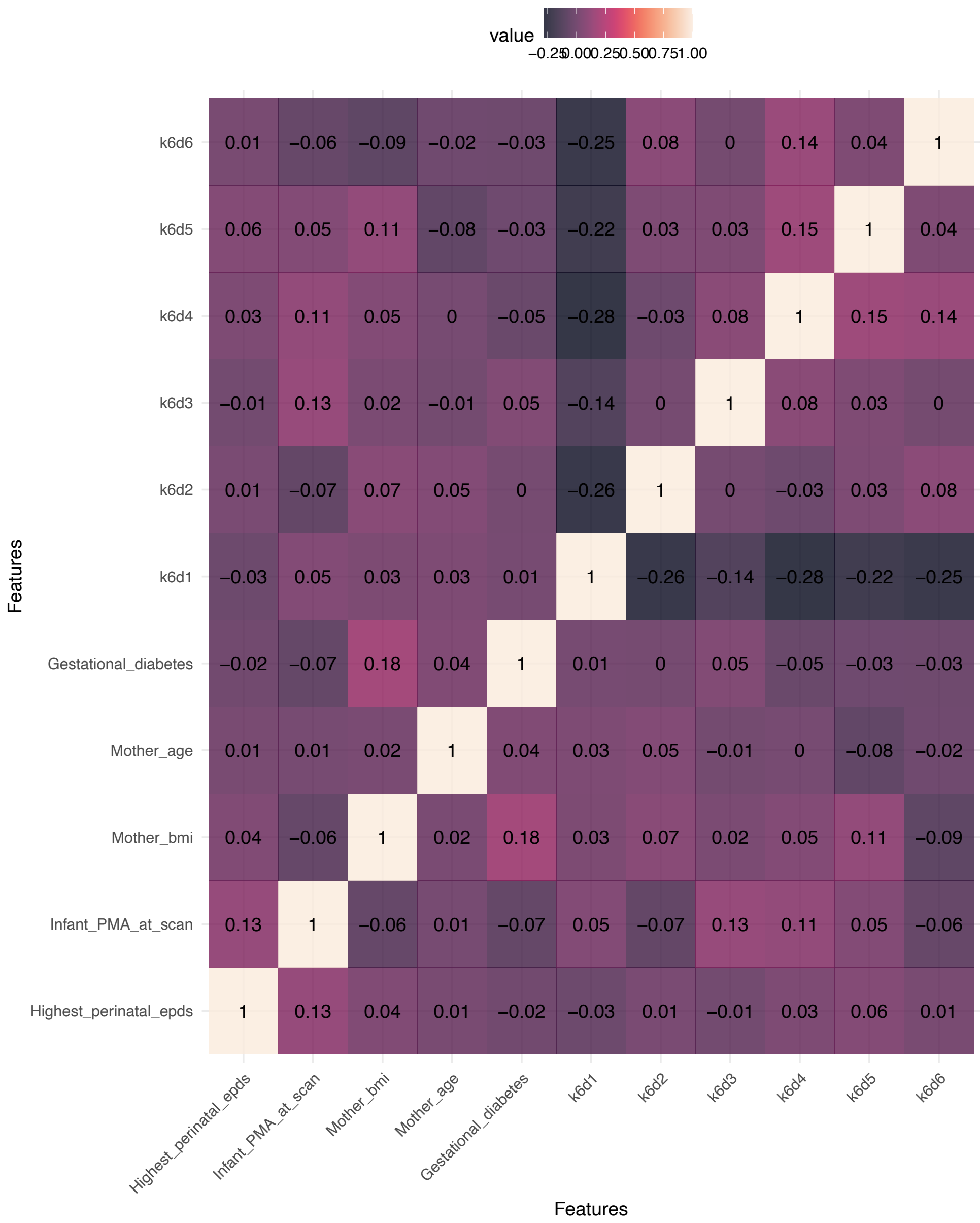


**Figure S6. Bivariate correlation coefficients between variables of interest and lifetimes of brain states (K = 6).** *Pearson's bivariate correlation coefficient is reported. k6d1- k6d6, lifetime of brain state #1–#6 when K = 6; Mother_bmi, maternal pre-pregnancy BMI; Infant_PMA_at_scan, infant postmenstrual age at scan (in weeks); Highest_perinatal_epds, maternal perinatal depressive symptoms.*

### 3.3 Transitions

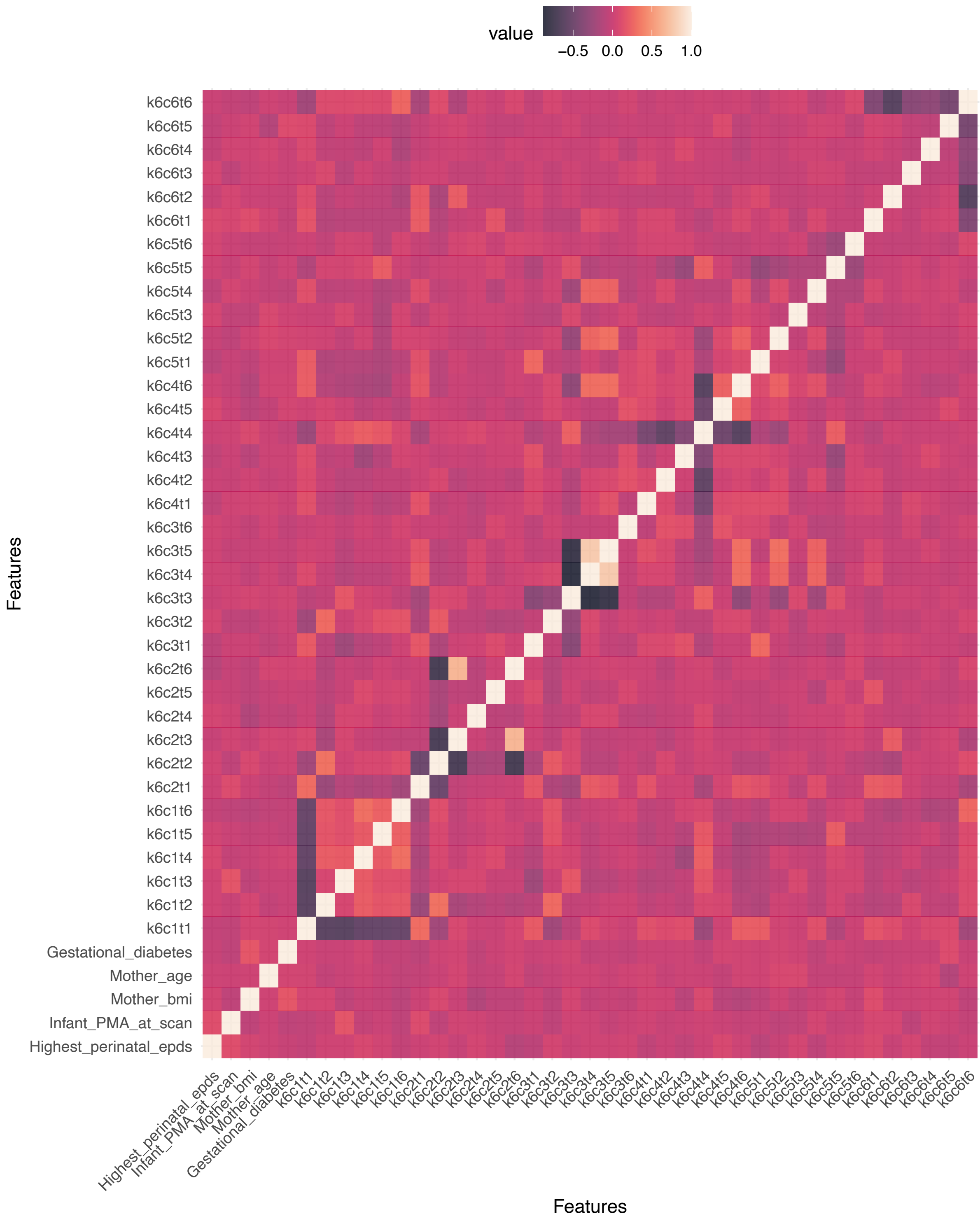


**Figure S7. Bivariate correlation matrix between the variables of interest and the probability of transition between brain states (K = 6).** *Pearson's bivariate correlation coefficient is reported. k6c1t2 - k6c6t6, transition probability from brain state #1 - #6 when K = 6; Mother_bmi, maternal pre-pregnancy BMI; Infant_PMA_at_scan, infant postmenstrual age at scan (in weeks); Highest_perinatal_epds, maternal perinatal depressive symptoms.*

## 4. Maternal prenatal BMI

### 4.1 Neonates' Frontoparietal network

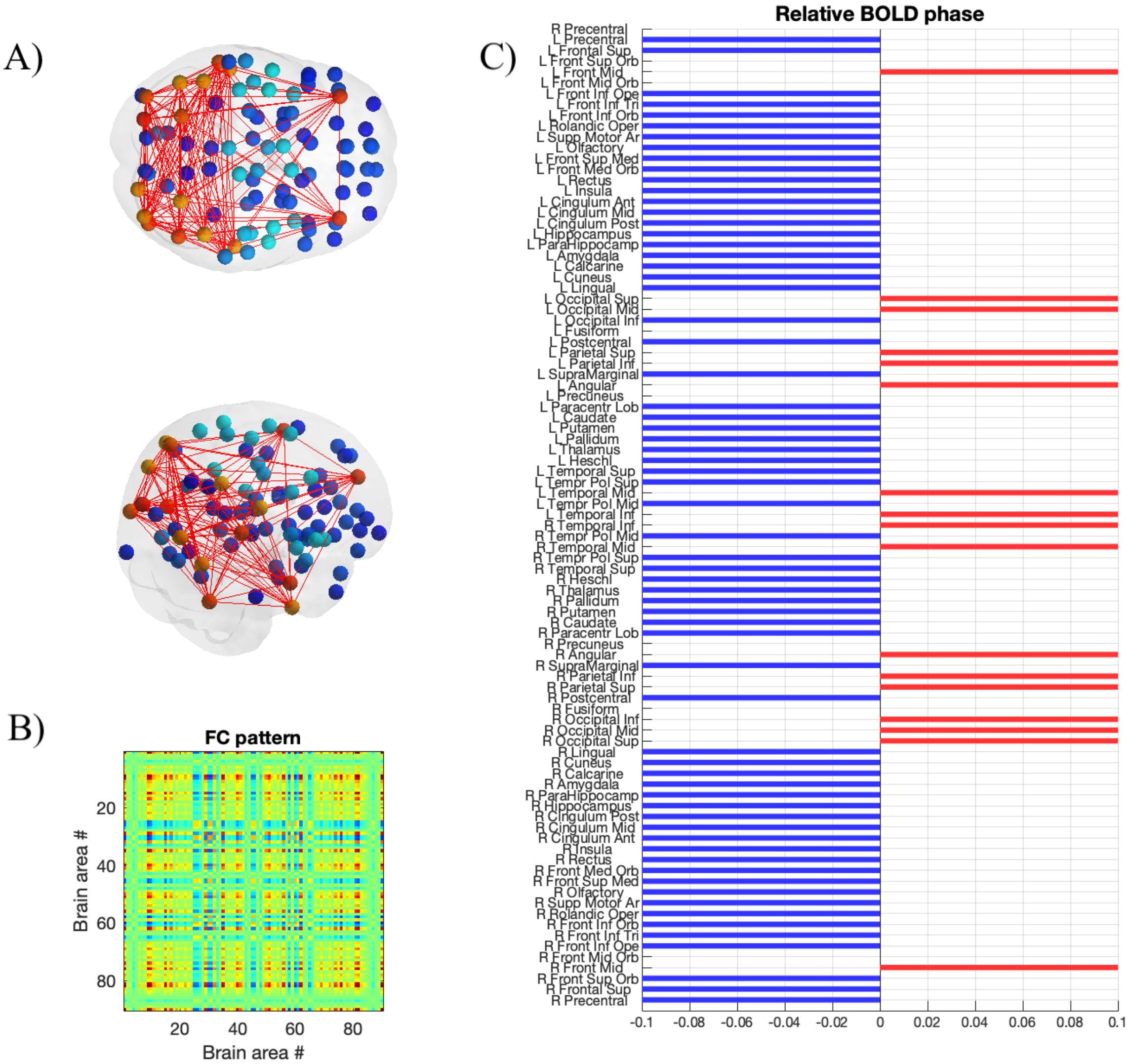


***Figure S8. Neonates' frontoparietal network.*** *The frontoparietal network (brain state 6 when K=6): A) network plot, B) the connectivity pattern in AAL atlas (90x90 brain areas), C) visualization of the brain areas that constitute the network (in red colour). The red brain areas form the brain network of interest (A), yellow-red-cells demarcate brain regions in 90 x 90 region-wise connectome matrix of the AAL atlas (B), and red bars in bar plots indicate the individual brain areas that are part of the network (C).*

### 4.2 Maternal prenatal BMI and brain state probability of occurrence (k = 6)

Maternal pre-pregnancy BMI was negatively associated with the probability of occurrence (i.e., the dominance of activity of an FC state) of FP functional network (i.e., brain state #6) which encompass superior frontal, superior parietal, and temporal brain regions (ß = -.129, *p* = .006) (Figure 2 of the main file). This association was of modest effect size but robust to the included covariates such as infant postmenstrual age at scan, sex, motion during scan, as well as maternal age and gestational diabetes. Below we report the scatter plots showing associations between maternal pre-pregnancy BMI and the probability of occurrence of all the brain states.

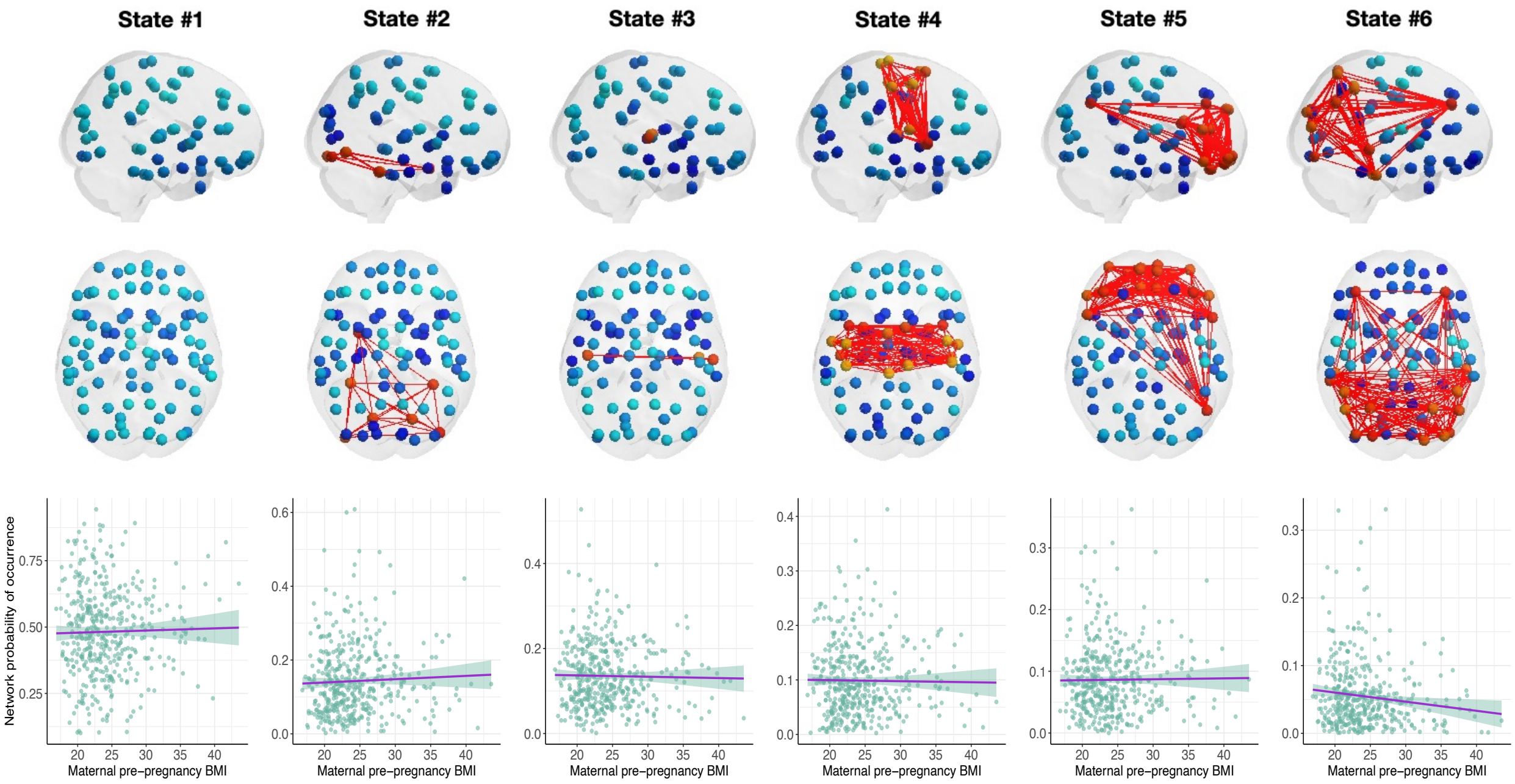


***Figure S9. Probability of occurrence of neonates' brain states and maternal pre-pregnancy BMI.*** *Repertoire of FN states assessed with LEiDA and association to maternal pre-pregnancy BMI (K = 6). The link between maternal pre-pregnancy BMI and the probability of occurrence of brain state #6 – the frontoparietal network- resulted statistically significant.*

### 4.3 Exploring the interaction between maternal prenatal BMI and perinatal depressive symptoms in predicting brain state probability of occurrence (k = 6)

Although beyond the primary scope of this study, we explored whether maternal pre-pregnancy BMI and depressive symptoms interact in shaping FP network occurrence. These results did not survive correction for multiple comparisons and are reported in the below.

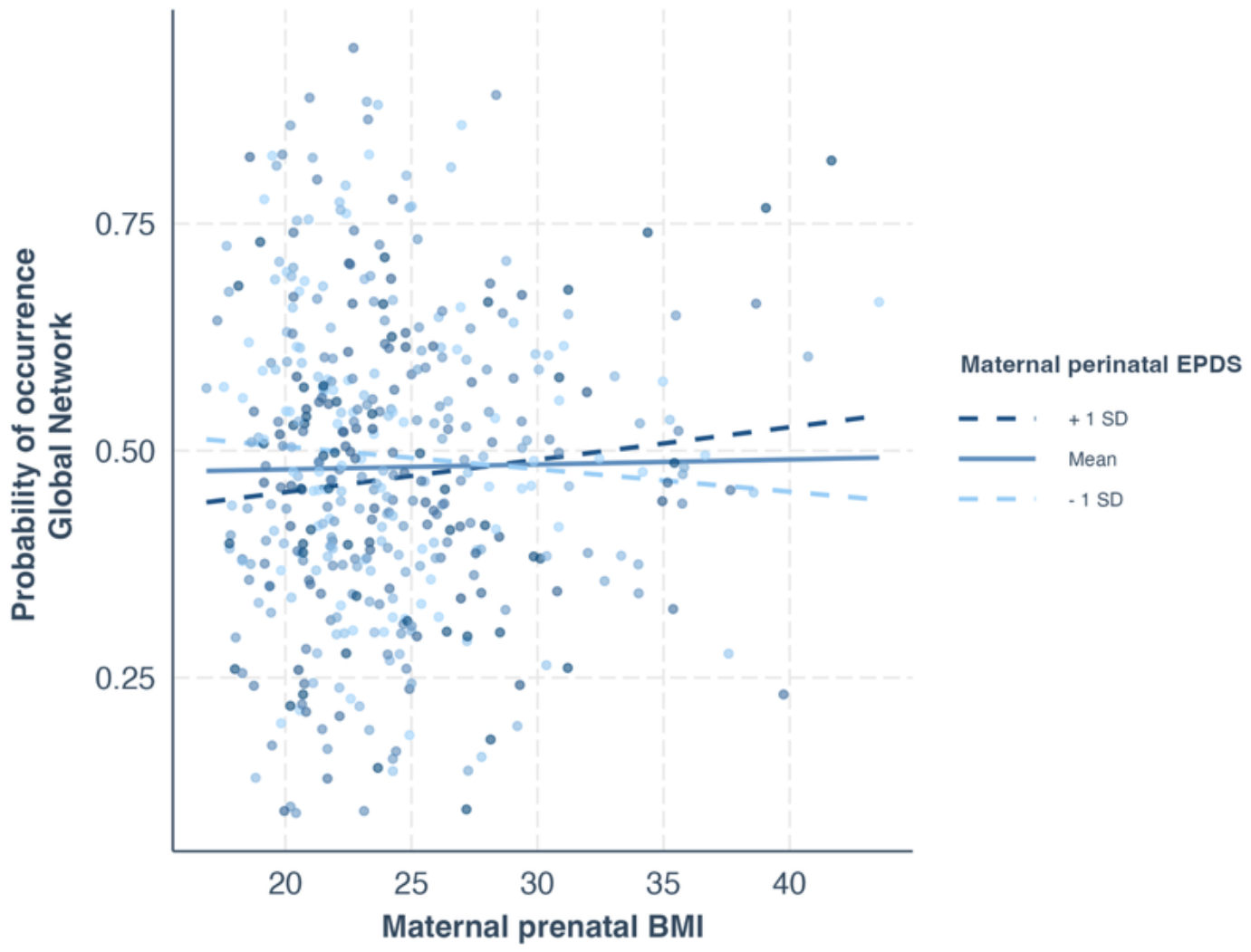


***Figure S10. Interaction between maternal prenatal BMI and perinatal depressive symptoms in predicting brain state #1 probability of occurrence (K = 6).*** *EPDS; Maternal perinatal depressive symptoms, BMI; Body Mass Index.*

| Term | Estimate | Std.Error | *p* | *p_Bonf* |
|---|---|---|---|---|
| Maternal perinatal EPDS | -0.0199 | 0.0083 | 0.016 | 0.134 |
| Maternal BMI | -0.0034 | 0.0027 | 0.203 | 1.000 |
| Maternal age | <0.0001 | 0.0017 | 0.996 | 1.000 |
| Gestational diabetes | 0.0156 | 0.0357 | 0.663 | 1.000 |
| Infant sex | -0.0403 | 0.0158 | 0.011 | 0.088 |
| Infant PMA at scan (weeks) | -0.0023 | 0.0048 | 0.640 | 1.000 |
| Motion | -0.0003 | 0.0001 | 0.034 | 0.276 |
| Maternal perinatal EPDS * Maternal BMI | 0.0007 | 0.0003 | 0.031 | 0.254 |

***Table S2. Interaction Model Results: maternal prenatal BMI and perinatal depressive symptoms predict the probability of occurrence of the brain state #1.*** *The table includes unstandardized coefficients (Estimate), standard errors (SE), uncorrected (p) and corrected (p_Bonf) p-values. EPDS; Maternal perinatal depressive symptoms, BMI; Body Mass Index, PMA; infant postmenstrual age at scan (in weeks), Motion; infants motion during scan.*

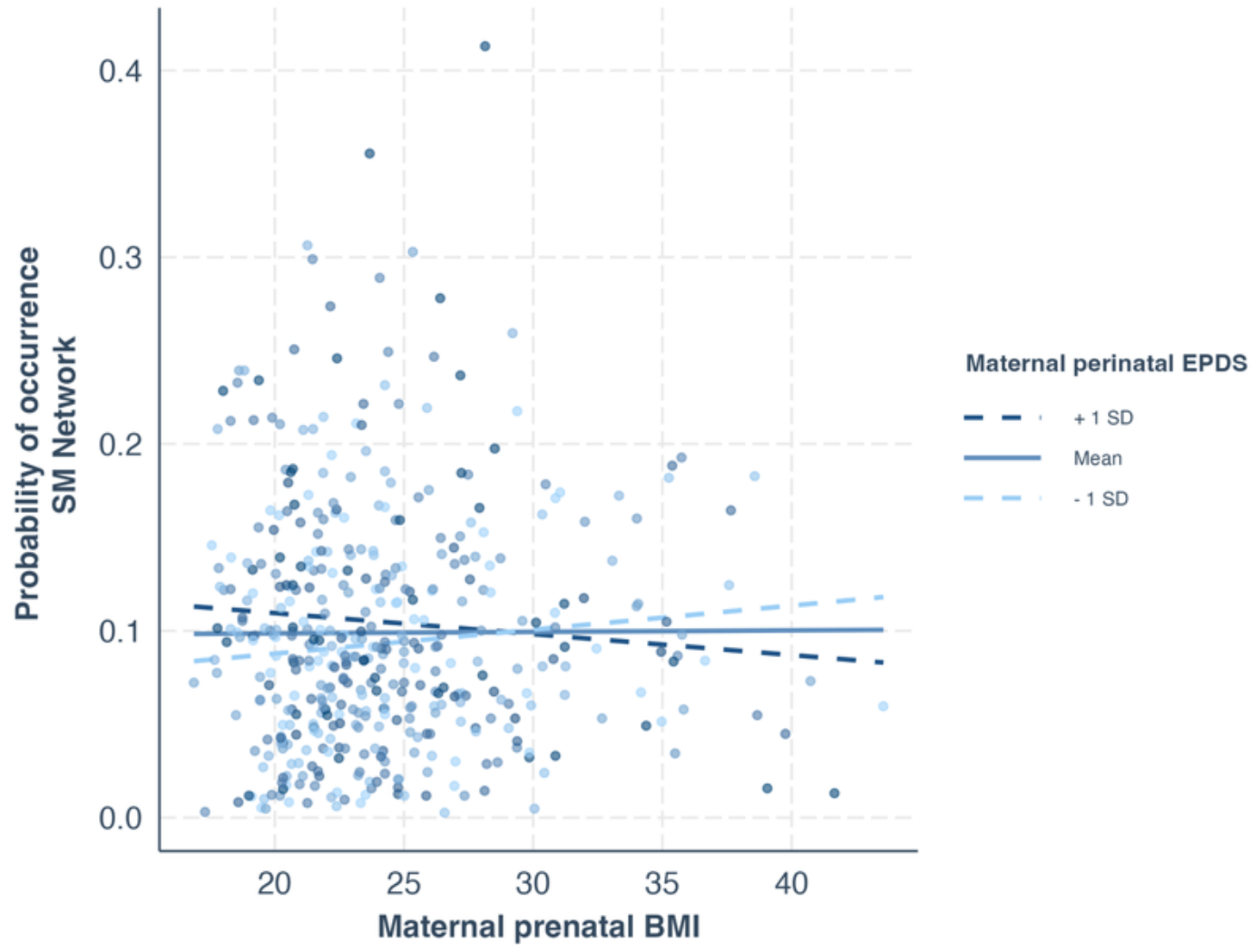


***Figure S11. Interaction between maternal prenatal BMI and perinatal depressive symptoms in predicting brain state #4 probability of occurrence.*** *SM; sensorimotor brain state (i.e., brain state #4); EPDS, Maternal perinatal depressive symptoms; BMI, Body Mass Index.*

| Term | Estimate | Std.Error | *p* | *p_Bonf* |
|---|---|---|---|---|
| Maternal perinatal EPDS | 0.0081 | 0.0033 | 0.013 | 0.109 |
| Maternal BMI | 0.0016 | 0.0010 | 0.118 | 0.946 |
| Maternal age | 0.0005 | 0.0007 | 0.453 | 1.000 |
| Gestational diabetes | -0.0167 | 0.0141 | 0.237 | 1.000 |
| Infant sex | 0.0035 | 0.0062 | 0.573 | 1.000 |
| Infant PMA at scan (weeks) | 0.0031 | 0.0019 | 0.099 | 0.747 |
| Motion | <0.0001 | 0.0001 | 0.843 | 1.000 |
| Maternal perinatal EPDS * Maternal BMI | -0.0003 | 0.0001 | 0.037 | 0.245 |

***Table S3. Interaction Model Results: maternal prenatal BMI and perinatal depressive symptoms predict the probability of occurrence of the sensorimotor state.*** *The table includes unstandardized coefficients (Estimate), standard errors (SE), uncorrected (p) and corrected (p_Bonf) p-values. EPDS; Maternal perinatal depressive symptoms, BMI; Body Mass Index, PMA; infant postmenstrual age at scan (in weeks), Motion; infants motion during scan.*

## 4.4 Maternal prenatal BMI and the probability of occurrence of the frontoparietal network

Maternal pre-pregnancy BMI was negatively associated with the probability of occurrence of FP functional network (i.e., brain state #6) encompassing superior frontal, superior parietal, and temporal brain regions (β = -0.129, $p = 0.006$) (Figure 2). This association was of modest effect size but robust to the included covariates (i.e., infant's PMA at scan, sex, motion during scan, maternal age, and gestational diabetes). Importantly, this result remained statistically significant after correcting for multiple comparisons using the Bonferroni correction, with the corrected p-value ($p_{Bonf} = 0.036$). It is worth mentioning that we observed the same association also when more brain states were defined. Specifically, when K was set to 7, 8, and 10, the association between the FP network and maternal pre-pregnancy BMI remained statistically significant (Tables S3, S4, and S5).

We report below standardized coefficients, uncorrected and corrected p-values for each FP states in different K solutions:

- brain state #7 for K = 7 (β = -0.132, $p = 0.006$, $p_{Bonf} = 0.041$)
- brain state #8 for K = 8 (β = -0.130, $p = 0.006$, $p_{Bonf} = 0.048$)
- brain state #10 for K = 10 (β = -0.133, $p = 0.005$, $p_{Bonf} = 0.037$)

| Term | Estimate | Std. Error | *p* |
|---|---|---|---|
| Maternal perinatal EPDS | 0.0005 | 0.0005 | 0.298 |
| Maternal BMI | -0.0014 | 0.0005 | 0.005 |
| Maternal age | <0.0001 | 0.0005 | 0.991 |
| Gestational diabetes | -0.0033 | 0.0100 | 0.739 |
| Infant sex | 0.0076 | 0.0044 | 0.088 |
| Infant PMA at scan (weeks) | -0.0024 | 0.0013 | 0.072 |
| Motion | 0.0002 | 0.0000 | <0.0001 |

***Table S4. Maternal prenatal BMI and frontoparietal network with K = 7.*** *The table includes unstandardized coefficients (Estimate), standard errors (SE), uncorrected p-values (p). EPDS; Maternal perinatal depressive symptoms, BMI; Body Mass Index, PMA; infant postmenstrual age at scan (in weeks), Motion; infants motion during scan.*

| Term | Estimate | Std. Error | *p* |
|---|---|---|---|
| Maternal perinatal EPDS | 0.0005 | 0.0005 | 0.2859 |
| Maternal BMI | -0.0013 | 0.0005 | 0.0067 |
| Maternal age | 0.0000 | 0.0005 | 0.9896 |
| Gestational diabetes | -0.0025 | 0.0099 | 0.7979 |
| Infant sex | 0.0077 | 0.0044 | 0.0810 |
| Infant PMA at scan (weeks) | -0.0019 | 0.0015 | 0.2105 |
| Motion | 0.0002 | 0.0000 | <0.0001 |

***Table S5. Maternal prenatal BMI and the frontoparietal network with K = 8.*** *The table includes unstandardized coefficients (Estimate), standard errors (SE), uncorrected p-values (p). EPDS; Maternal perinatal depressive symptoms, BMI; Body Mass Index, PMA; infant postmenstrual age at scan (in weeks), Motion; infants motion during scan.*

| Term | Estimate | Std. Error | *p* |
|---|---|---|---|
| Maternal perinatal EPDS | 0.0005 | 0.0005 | 0.2859 |
| Maternal BMI | -0.0013 | 0.0005 | 0.0067 |
| Maternal age | 0.0000 | 0.0005 | 0.9896 |
| Gestational diabetes | -0.0025 | 0.0099 | 0.7979 |
| Infant sex | 0.0077 | 0.0044 | 0.0810 |
| Infant PMA at scan (weeks) | -0.0019 | 0.0015 | 0.2105 |
| Motion | 0.0002 | 0.0000 | < 0.0001 |

***Table S6. Maternal prenatal BMI and the frontoparietal network with K = 10.*** *The table includes unstandardized coefficients (Estimate), standard errors (SE), uncorrected p-values (p). EPDS; Maternal perinatal depressive symptoms, BMI; Body Mass Index, PMA; infant postmenstrual age at scan (in weeks), Motion; infants motion during scan.*

**4.5 Maternal prenatal BMI and Lifetime**

Dwell times, also called lifetime, of brain states #5 (ß = .112, *p* = .011, $p_{Bonf}$ = 0.077) and #6 (ß = -.084, *p* = .044, $p_{Bonf}$ = 0.310) resulted significantly predicted by maternal pre-pregnancy BMI with the infant's gestational age (GA) at scan, sex, motion during scan, as well as maternal age and gestational diabetes as covariates. No results survived Bonferroni correction for multiple comparison.

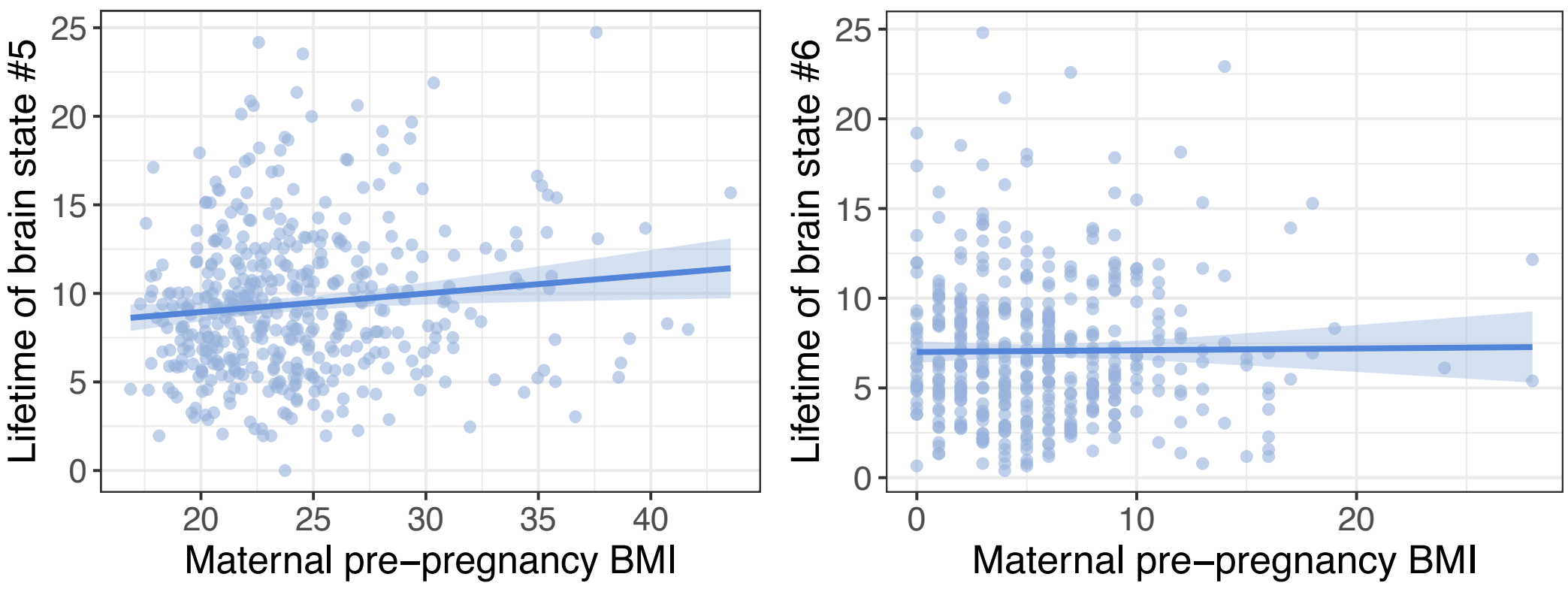


***Figure S12. Lifetime and maternal pre-pregnancy BMI (K = 6).*** *Higher maternal pre-pregnancy BMI positively predicts the time spent in functional connectivity state #5 and negatively predicts the time spent in functional connectivity state #6 when K = 6.*

**5. Sensitivity**

We conducted several sensitivity analyses to test the robustness of the association between maternal pre-pregnancy BMI and the probability of occurrence of the Frontoparietal (FP) network. First, we excluded preterm-born infants (N = 33) from the sample. Results confirmed the negative association (β = -0.14, $p = 0.005$; Table S7). Importantly, this association remained statistically significant after correcting for multiple comparisons using the Bonferroni correction ($p_{Bonf} = 0.038$). These findings suggest that the relationship between maternal pre-pregnancy BMI and the FP network is not driven by the inclusion of preterm infants.

| Term | Estimate | Std.Error | *p* |
|---|---|---|---|
| Maternal perinatal EPDS | 0.0002 | 0.0005 | 0.713 |
| Maternal BMI | -0.0014 | 0.0005 | 0.005 |
| Maternal age | 0.0001 | 0.0005 | 0.840 |
| Gestational diabetes | -0.0023 | 0.0102 | 0.738 |
| Infant sex | 0.0087 | 0.0046 | 0.059 |
| Infant PMA at scan (weeks) | -0.0039 | 0.0014 | 0.064 |
| Motion | 0.0002 | <0.001 | <0.001 |

***Table S7. Main model results excluding preterm infants.*** *The table includes unstandardized coefficients (Estimate), standard errors (SE) and uncorrected p-values (p). EPDS; Maternal perinatal depressive symptoms, BMI; Body Mass Index, PMA; infant postmenstrual age at scan (in weeks), Motion; infants motion during scan.*

Second, we conducted a sensitivity analysis including GA at birth as a coviariate. The results confirmed the negative association (β = -0.14, $p = 0.006$; Table S8) which remained statistically significant after correcting for multiple comparisons ($p_{Bonf} = 0.048$). These findings suggest that the relationship between maternal pre-pregnancy BMI and the FP network is not driven by GA at birth.

| Term | Estimate | Std.Error | *p* |
|---|---|---|---|
| Maternal perinatal EPDS | 0.0005 | 0.0005 | 0.362 |
| Maternal BMI | -0.0014 | 0.0005 | 0.006 |
| Maternal age | 0.0001 | 0.0005 | 0.901 |
| Gestational diabetes | -0.003 | 0.0104 | 0.771 |
| Infant sex | 0.0092 | 0.0046 | 0.047 |
| Infant PMA at scan (weeks) | -0.0032 | 0.0016 | 0.038 |
| Motion | 0.0002 | <0.001 | <0.001 |
| Infant GA at birth (weeks) | 0.0013 | 0.0014 | 0.350 |

***Table S8. Main model results including gestational age as covariate.*** *The table includes unstandardized coefficients (Estimate), standard errors (SE) and uncorrected p-values (p). EPDS; Maternal perinatal depressive symptoms, BMI; Body Mass Index, PMA; infant postmenstrual age at scan (in weeks), Motion; infants motion during scan.*

Third, we conducted a sensitivity analysis testing the association between maternal pre-pregnancy BMI and the probability of occurrence of the FP network, including maternal medication use during pregnancy as a covariate. The results confirmed the negative association (β = -0.14, $p = 0.006$, $p_{Bonf} = 0.04$; Table S9).

| Term | Estimate | Std.Error | *p* |
|---|---|---|---|
| Maternal perinatal EPDS | 0.0004 | 0.0005 | 0.362 |
| Maternal BMI | -0.0014 | 0.0005 | 0.006 |
| Maternal age | 0.0001 | 0.0005 | 0.985 |
| Gestational diabetes | -0.0024 | 0.0104 | 0.811 |
| Infant sex | 0.0091 | 0.0046 | 0.048 |
| Infant PMA at scan (weeks) | -0.0024 | 0.0014 | 0.082 |
| Motion | 0.0002 | <0.001 | <0.001 |
| Maternal medications during pregnancy | -0.0026 | 0.0019 | 0.172 |

***Table S9. Main model results including maternal medication use during pregnancy as covariate.*** *The table includes unstandardized coefficients (Estimate), standard errors (SE) and uncorrected p-values (p). EPDS; Maternal perinatal depressive symptoms, BMI; Body Mass Index, PMA; infant postmenstrual age at scan (in weeks), Motion; infants motion during scan.*

Finally, we controlled the model for the possible effect of maternal smoking (N = 11) during pregnancy. The results confirmed the negative association (β = -0.13, $p = 0.006$, $p_{Bonf} = 0.04$; Table S10).

| Term | Estimate | Std.Error | *p* |
|---|---|---|---|
| Maternal perinatal EPDS | 0.0004 | 0.0005 | 0.372 |
| Maternal BMI | -0.0013 | 0.0005 | 0.006 |
| Maternal age | 0.0003 | 0.0005 | 0.947 |
| Gestational diabetes | -0.0035 | 0.0104 | 0.737 |
| Infant sex | 0.0094 | 0.0046 | 0.042 |
| Infant PMA at scan (weeks) | -0.0025 | 0.0014 | 0.064 |
| Motion | 0.0002 | <0.001 | <0.001 |
| Smoking | -0.0002 | 0.0054 | 0.965 |

***Table S10. Main model results including smoking during pregnancy as covariate.*** *The table includes unstandardized coefficients (Estimate), standard errors (SE) and uncorrected p-values (p). EPDS; Maternal perinatal depressive symptoms, BMI; Body Mass Index, PMA; infant postmenstrual age at scan (in weeks), Motion; infants motion during scan.*

Overall, these sensitivity analyses showed that the main effect of maternal pre-pregnancy BMI on the probability of occurrence of the FP network was not driven by any of the

confounders considered (i.e., preterm birth, GA at birth, maternal medication use, and smoking during pregnancy), further supporting the robustness of our main findings.

**References**


Alonso S., Tyborowska A., et al. Depression recurrence is accompanied by longer periods in default mode and more frequent attentional and reward processing dynamic brain-states during resting-state activity. Hum. Brain Mapp. 44, 26475 (2023). https://doi.org/10.1002/hbm.26475

Boashash B. Estimating and interpreting the instantaneous frequency of a signal. I. Fundamentals. Proc. IEEE 80, 520–538 (1992). https://doi.org/10.1109/5.135376

Cabral J. et al. Cognitive performance in healthy older adults relates to spontaneous switching between states of functional connectivity during rest. Sci. Rep. 7, 5135 (2017).

Vidaurre D. et al. Spectrally resolved fast transient brain states in electrophysiological data. NeuroImage 126, 81–95 (2016). https://doi.org/10.1016/j.neuroimage.2015.11.047

Vohryzek J, Deco G, Cessac B, Kringelbach ML, Cabral J. Ghost attractors in spontaneous brain activity: recurrent excursions into functionally-relevant BOLD phase-locking states. Front. Syst. Neurosci. 14, 20 (2020). https://doi.org/10.3389/fnsys.2020.00020
